# An ethnobotanical survey of medicinal plants used in the East Sepik province of Papua New Guinea

**DOI:** 10.1186/s13002-015-0065-8

**Published:** 2015-11-14

**Authors:** Michael Koch, Dickson Andrew Kehop, Boniface Kinminja, Malcolm Sabak, Graham Wavimbukie, Katherine M. Barrows, Teatulohi K. Matainaho, Louis R. Barrows, Prem P. Rai

**Affiliations:** Center for Biopharmaceutical Research, De La Salle Health Sciences Institute, Dasmarinas, Cavite Philippines; School of Medicine and Health Sciences, University of Papua New Guinea, PO Box 5623, Boroko, NCD Papua New Guinea; Department of Pharmacology and Toxicology, University of Utah, 30 S. 2000 E., Salt Lake City, 84112 UT USA

**Keywords:** Papua New Guinea, East Sepik, Medicinal plants, Bougainville, Eastern highlands, Quantitative ethnopharmacology

## Abstract

**Background:**

Rapid modernization in the East Sepik (ES) Province of Papua New Guinea (PNG) is resulting in a decrease in individuals knowledgeable in medicinal plant use. Here we report a synthesis and comparison of traditional medicinal plant use from four ethnically distinct locations in the ES Province and furthermore compare them to two other previous reports of traditional plant use from different provinces of PNG.

**Methods:**

This manuscript is based on an annotated combination of four Traditional Medicines (TM) survey reports generated by University of Papua New Guinea (UPNG) trainees. The surveys utilized a questionnaire titled “Information sheet on traditional herbal preparations and medicinal plants of PNG”, administered in the context of the TM survey project which is supported by WHO, US NIH and PNG governmental health care initiatives and funding. Regional and transregional comparison of medicinal plant utilization was facilitated by using existing plant databases: the UPNG TM Database and the PNG Plant Database (PNG Plants) using Bayesian statistical analysis.

**Results:**

Medicinal plant use between four distinct dialect study areas in the ES Province of PNG showed that only a small fraction of plants had shared use in each area, however usually utilizing different plant parts, being prepared differently and to treat different medical conditions. Several instances of previously unreported medicinal plants could be located. Medicinally under- and over-utilized plants were found both in the regional reports and in a transregional analysis, thus showing that these medicinal utilization frequencies differ between provinces.

**Conclusions:**

Documentation of consistent plant use argues for efficacy and is particularly important since established and effective herbal medicinal interventions are sorely needed in the rural areas of PNG, and unfortunately clinical validation for the same is often lacking. Despite the existence of a large corpus of medical annotation of plants for PNG, previously unknown medical uses of plants can be uncovered. Furthermore, comparisons of medicinal plant utilization is possible if databases are reformatted for consistencies that allow comparisons. A concerted effort in building easily comparable databases could dramatically facilitate ethnopharmacological analysis of the existing plant diversity.

## Background

Papua New Guinea (PNG) is a largely rural country characterized by at least 800 ethnic traditions dispersed over 462,840 km^2^ [1, 2]. Most of the population resides in small villages, situated in diverse environs that range from montane rainforest to lowland river deltas and small tropical islands. Settled 49,000–44,000 years ago (Ivane Valley in the PNG Highlands) [3], PNG is blessed with extraordinary biological diversity and a rich but fragmented cultural tapestry of customs, art, spiritual beliefs and medicinal knowledge.

The East Sepik Province is situated in the northwest of the country bordered by the West Sepik Province (West), Madang Province (East), the Bismark Sea (North) and Enga Province (South). East Sepik (43,426 km^2^) is characterized by mountainous terrain to the south and west and the costal floodplain of the Sepik river, which flows west to east through the province [4]. The approximately 350,000 inhabitants have to rely on 37 health centers for provisioning health care and heavily supplement western medicines with traditional medicines (TM) [4, 5]. The 10 % mortality rate for children under 5 years reflects the difficulty of providing adequate health care in the East Sepik Province. In an effort to supplement health care with effective traditional medicins the University of Papua New Guinea (UPNG), endorsed by the PNG government, struck a collaboration with the WHO to develop reliable traditional medicines (TM) and safe practices (outlined in the 2001–2010 PNG National Health Plan [6]). Part of this project includes traditional medicine surveys performed by UPNG students working in their kinship (“wantok”) communities. The data are then recorded in a proprietary database maintained at UPNG [7]. This database serves as central repository for PNG traditional medicine practices, preserving cultural traditions from many diverse communities.

## Methods

The TM surveys are performed by UPNG students who are instructed on plant identification, preservation, herbal medicine use, and trained on how to administer the survey instrument entitled “Information sheet on traditional herbal preparations and medicinal plants of Papua New Guinea.” The survey questionnaire is the basis for semi-structured face-to-face interviews with healers, herbalists, birth attendants, and bone setters. Field vouchers of medicinal plants (twigs with leaves, fruits, flowers, nuts, etc.) are harvested under guidance of the healer and dried and compressed in newspapers. Photographs, descriptions and the pressed plant samples are assigned a voucher number and deposited with the UPNG Herbarium for later identification and reference [8].

The data concerning plant use are written up under supervision into student authored reports and the plant information is entered into the UPNG Traditional Medicines Database, which contains the combined data from reports generated by a decade’s work in this endeavor. It is the student reports that provide the base information for this current report.

Four student reports from the East Sepik Province representing four distinct language dialect communities have been compiled here: “Traditional Medicinal Plants and Practices in the Waskuk Hills Area of Ambunti District in East Sepik (2005)” by Dickson Andrew Kehop; “Traditional Medicine Practices in Niungweko and Kunjingini (MUL) Area of Wosera-Gawi District in East Sepik (2006)” by Boniface Kinminja; “Enthnobotanical Survey of Traditional Medicine in East Yangoru, East Sepik Province, Papua New Guinea” (2004) by Graham Wavimbukie; and “Traditional Medicinal Plants and Practices in Kairiru Island East Sepik Province Papua New Guinea (2004) by Malcolm Sabak The first three reports (DK, BK and GW, respectively) are inland above the Sepik floodplain at elevations ranging from 150 to 300 m. The report from Kairiru Island is referred to as MS. The specific village communities interviewed included: Bangus and Mariawai villages (DK), Niungweko and Mul (Kunjingini 1) villages (BK); Marambanja, Saina, Ambukanja, Parina, Jawia, Mandien, Bukiendoun, Sausenduon, Hangrerak and Kiarivu villages (GW) and Rumlal, Shagur and Bou villages (MS).

A compilation of references for medicinal plants described from Papua New Guinea is currently in progress (50 references) in our lab. These references were used to determine if plants collected in the current East Sepik survey work have been previously reported for medicinal use. Comparison of this East Sepik medicinal plant report to our previous reports from Bougainville [8] and the Eastern Highlands [9] was accomplished after editing the previous two reports to match the current format, including codes for conditions treated.

Overall flora distribution data for PNG was obtained for the provinces of East Sepik, Eastern Highlands, and the autonomous region of Bougainville from the PNG Plants Database [10]. The data was imported into Microsoft Excel™, formatted and edited as necessary, then processed with standard Unix (Linux) utilities to produce a formatted list containing the plant family, genus and species (if known). Duplicated instances of plants in the list were removed. The family names were split off, sorted and processed using a Python script on a Raspberry Pi Model B (http://www.raspberrypi.org) to quantify the number of instances of each plant family cited in the list. In general, when multiple names for the same species were found, we attempted to match plant family names to the PNG Plant Database versions to allow for quantitative comparison. Statistical analysis was carried out exactly as previously described by Weckerle et al. [11] using the “beta.inv” function provided in Microsoft Excel™ to calculate the 95 % inferior and superior credible intervals for the data. Comparison of the currently reported East Sepik medical plants to the Traditional Medicines Plant Database maintained at UPNG was carried out similarly. Discrepancies in plant family names were resolved as described above, the family names were adapted to the family names in the UPNG Traditional Medicines Database; resulting in a slightly different number of plant families for the traditional plant uses reported.

Plant families which are considered overused have an inferior credible interval that lies above the superior credible interval for the regional total data. Plant families that are considerend underused have a superior credible interval below the inferior credible interval of the regional total data set distribution.

## Results and discussion

### East Sepik reports

The combined student reports contained 299 entries (including unidentified plants (*n* = 6)) from the East Sepik province of PNG. The reports collated 205 plants, of which 139 were identified to species and 66 to genus, from a total of 71 families. Three reports were from areas of estimated 150 m to 300 m elevation (DK, BK and GW voucher numbers), DK from Waskuk Hills in the center of Sepik province, and BK and GW from elevated areas of the province north of the Sepik river plain and southwest of the capital Wewak. One report (MS voucher numbers) came from an island (Kairiru Island) situated close to the coast of East Sepik. The combined dataset is presented in Table 1 for all four areas of the East Sepik province under consideration.

**Table 1:** Plants reported as medicinally used in 4 study areas in East Sepik Province

Voucher	Plant ID^References^	Family	Local Name	Ailment	AilmentCode	PartCode	PrepCode	RouteCode
MS 02/04	*Abelmoschus manihot* (L.) Medik [32–39]	Malvaceae	Wasniat	Uterine contraction	REP	L	D	O
GW 05/04	*Acalypha grandis* Benth [33, 40]	Euphorbiaceae	Unknown	Antidote to poisoning (Chemical or acid)	POIS	L	S	O
DK 16/05	*Acalypha* sp. [7, 33–35, 39–49]	Euphorbiaceae	Mikirme	Malaria	MAL	L	D	O
GW 88/04	*Acalypha* sp. [7, 33–35, 39–49]	Euphorbiaceae	Winghongong	Cough, shortness of breath	RESP	Sap	S	O
BK 057/06	*Acalypha wilkesiana* Müll. Arg [42, 44, 45, 47–49]	Euphorbiaceae	Polembieri	Cough, shortness of Breath	RESP	L	D	O
DK 38/05	*Ageratum conyzoides* (L.) L [8]	Asteraceae	Mungrimb	Sore	SKIN	L	R	T
GW 56/04	*Aglaia* sp. [33, 50, 51]	Meliaceae	Waniembri	Fevers, malaria	FEV/MAL	L	B	I
GW 09/04	*Albizia procera* (Roxb.) Benth [34]	Fabaceae	He’re	Malaria, pneumonia, asthma	MAL/RESP	B	S	I & O
BK 058/06	*Albizia saman* (Jacq.) Merr [34, 52]	Mimosaceae	Yundimi	Induce sleep	PSYCH	L	D	T
DK 08/05	*Allophylus cobbe* (L.) Raeusch [8, 42, 51, 53]	Sapindaceae	Haim	Scabies	SKIN	B	C	O
GW 50/04	*Allophylus cobbe (L.)* Raeusch [8, 42, 51, 53]	Sapindaceae	Wah	Skin pox, cough	SKIN/RESP	L	D	T | O
DK 37/05	*Alocasia cucullata* (Lour.) G. Don	Araceae	Waken	Boil	SKIN	Root	R	T
MS 07/04	*Alocasia* sp. [8, 33–35, 43, 50, 51]	Araceae	Waiyat	Abortion	REP	L	S	O
GW 27/04	*Alphitonia incana* (Roxb.) Teijsm. & Binn. ex Kurz [33–35, 43, 54]	Rhamnaceae	Hushu	Scabies	SKIN	B	S	T
GW 24/04	Alpinia sp. [8, 9, 33, 34, 42, 43, 55–57]	Zingiberaceae	Wambelekie	Cancer (mouth), hypertension	CANC/CV	R	D	O
MS 03/04	*Alpinia* sp. [8, 9, 33, 34, 42, 43, 55–57]	Zingiberaceae	Kasai	Cough	RESP	yShoot	S	O
MS 41/04	*Alpinia* sp. [8, 9, 33, 34, 42, 43, 55–57]	Zingiberaceae	Sinup	Fever, headache, body ache	FEV/HEAD/PAIN/SWELL	yShoot	S	O
MS 68/04	*Alpinia* sp. [8, 9, 33, 34, 42, 43, 55–57]	Zingiberaceae	Kasai	Antidepressant	PSYCH	L & yShoot	S	O
BK 022/06	*Alstonia scholaris* (L.) R.Br [7–9, 34, 39, 40, 42, 43, 46–49, 51–53, 57–64]	Apocynaceae	Kam-bh	Malaria, diarrhoea, asthma, sores	MAL/GAST/RESP/SKIN	L |Sap | Sap	D | D | S	O
DK 25/05	*Alstonia scholaris* (L.) R.Br [7–9, 34, 39, 40, 42, 43, 46–49, 51–53, 57–64]	Apocynaceae	Chimb	Scabies	SKIN	B	C	O
GW 16/04	*Alstonia scholaris* (L.) R.Br [7–9, 34, 39, 40, 42, 43, 46–49, 51–53, 57–64]	Apocynaceae	Hembe	Fever, malaria, cough, diarrhoea	FEV/MAL/RESP/GAST	Sap	S	O
MS 04/04	*Alstonia scholaris* (L.) R.Br [7–9, 34, 39, 40, 42, 43, 46–49, 51–53, 57–64]	Apocynaceae	Kaisabok	Fever, headache	FEV/HEAD	B	D	O
BK 034/06	*Amomum aculeatum* Roxb [39, 42, 53, 58, 65]	Zingiberaceae	Takkwa hamba	Asthma, scabies	RESP/SKIN	Stem	C	O & T
DK 19/05	*Amomum aculeatum* Roxb [39, 42, 53, 58, 65]	Zingiberaceae	Guinj Nikir	Fever	FEV	Whole	V	I
DK 53/05	*Angiopteris evecta* (G. Forst.) Hoffm [8, 56]	Marattiaceae	Yarchapa	Shortness of breath	RESP	Shoot & Root	S	O
MS 01/04	*Archidendron* sp. [8, 66]	Fabaceae	Niar	Diarrhoea, asthma, fever, headache	HEAD/FEV/GAST	B	D	O
DK 02/05	*Areca catechu* L [8, 34, 37, 39, 42, 43, 51, 67, 68]	Arecaceae	Maimb	Abdominal ache, whitespots	GAST/SKIN	Shoot | Nut	R | R	T | O
MS 10/04	*Aristolochia* sp. [8, 9, 34, 43, 48, 56, 59, 61, 63]	Aristolochiaceae	Mutamuth	Epigastric pain	GAST	L	R	T
MS 73/04	*Aristolochia* sp. [8, 9, 34, 43, 48, 56, 59, 61, 63]	Aristolochiaceae	War sapiau	Blocked nose, flu, cough	RESP	L	R	I
MS 23/04	*Artocarpus altilis* (Parkinson ex F.A. Zorn) Fosberg [8, 34, 43, 50, 61]	Moraceae	Kaikning	Hemorrhage	WOUND	Sap	R	O
GW 79/04	*Asclepias* sp.	Apocynaceae	Huaraloho	Enlarged spleen	ORG	Root	S	O
DK 21/05	*Asplenium nidus* L [8, 53]	Aspleniaceae	Yimangir	Infant back ache	CHILD	L	R	T
BK 039/06	*Averrhoa carambola* L.	Oxalidaceae	Macosembi	Aasthma, sore,fresh cut	RESP/SKIN/WOUND	Fruit	R | R	O | T
DK 01/05	*Averrhoa carambola* L.	Oxalidaceae	Waskapui	Cough	RESP	Fruit	D	O
MS 27/04	*Barringtonia asiatica* (L.) Kurz [38, 39, 42, 46, 51, 59, 64]	Lecythidaceae	Wut	Antipsychotic	PSYCH	B	D	O
GW 40/04	*Bidens pilosa* L. [7, 9, 32, 34–36, 39, 42, 43, 50, 57, 60]	Asteraceae	Miniesihaik	Eye infections, bleeding	INF/WOUND	Root	S	T
DK 11/05	*Bixa orellana* L [7, 35, 42, 43, 50, 53]	Bixaceae	Noksinu	Grille	SKIN	Seed	S	T
MS 53/04	*Breynia* sp. [7, 33, 34, 39, 42, 43, 50, 53, 58, 68, 69]	Phyllanthaceae	Smallak	Sore gums	DENT	yShoot	S	T
MS 61/04	*Breynia* sp. [7, 33, 34, 39, 42, 43, 50, 53, 58, 68, 69]	Phyllanthaceae	Murpopau	Fever, joint pain, headache (severe)	FEV/PAIN/HEAD	B	S	O
GW 21/04	*Bryophyllum pinnatum* (Lam.) Oken [7, 42, 51, 67]	Crassulaceae	Golip	Strong cough	RESP	L	D	O
MS 21/04	*Bryophyllum pinnatum* (Lam.) Oken [7, 42, 51, 67]	Crassulaceae	Mitultul	Ulcer	SKIN	L	H	T
BK 006/06	*Calamus* sp. [8, 33, 35, 43, 53, 57]	Arecaceae	Bal	Fever, headache, malaria, cough, malnutrition	FEV/HEAD/MAL/NUT	Sap	S	O
BK 051/06	*Calamus* sp. [8, 33, 35, 43, 53, 70]	Arecaceae	Gwalkipi	Dehydration	NUT	Sap	S	O
GW 92/04	*Calamus* sp. [8, 33, 35, 43, 53, 64]	Arecaceae	Peli	General cleansing	MAINT	Sap	S	O
MS 38/04	*Calamus* sp. [8, 33, 35, 43, 53, 64]	Arecaceae	War huk	Asthma	RESP	Sap	S	O
MS 85/04	*Callicarpa longifolia* Lam [34, 51]	Verbenaceae	Yeaik	Sore in baby’s mouth	CHILD	B	MS	T
MS 20/04	*Calophyllum inophyllum* L. [34, 39, 42, 43, 61, 67]	Guttiferae	Sabour	Toothache	DENT	B	D	O
MS 32/04	*Calotropis gigantea* (L.) (L.) Dryand [51]	Apocynaceae	Sasus	Fever, headache	FEV/HEAD	L	V	I
DK 56/05	*Campnosperma brevipetiolatum* Volkens [71]	Anacardiaceae	Gwart	Ulcer	SKIN	Sap	S	T
BK 010/06	*Campnosperma* sp.	Anacardiaceae	Biakuar	Sores, scabies, fresh cut, wound, hair and skin (as oil), removal of spear in skin	WOUND/SKIN	B	S	T
MS 39/04	*Canarium* sp. [34, 42, 43, 50, 57, 70]	Burseraceae	Klakul	Emetic	GAST	B	S	O
MS 64/04	*Canarium* sp. [34, 42, 43, 50, 57, 70]	Burseraceae	Yamuok	Ulcer	SKIN	Sap	S	T
DK 15/05	*Capsicum annuum* L.	Solanaceae	Seraimbsik	Malaria	MAL	Fruit & Seed	C	O
DK 34/05	*Carica papaya* L [42, 43, 46, 47, 59, 64, 68]	Caricaceae	Pous	Malaria	MAL	Root	D	O
DK 26/05	*Caryota mitis* Lour.	Arecaceae	Tosh	Shortness of Breath	RESP	Succus	S	O
MS 69/04	*Caryota rumphiana* Mart. [39, 53]	Arecaceae	Yamoun	Toothache	DENT	yShoot	M	O
BK 028/06	*Cascabela thevetia* (L.) Lippold [51]	Apocynaceae	Lai	Sores	SKIN	L & Seed	S	T
BK 044/06	*Cassia alata* L [7, 8, 34, 37, 38, 42, 46, 49, 52, 59, 61, 63, 67, 69]	Fabaceae	Yundilipgi	Grille and white spot	SKIN	L	R	T
DK 43/05	*Cassia alata* L [7, 8, 34, 37, 38, 42, 46, 49, 52, 59, 61, 63, 67, 69]	Fabaceae	Apkuaiamboi	Grille	SKIN	L	H | R	T
GW 01/04	*Cassia alata* L [7, 8, 34, 37, 38, 42, 46, 49, 52, 59, 61, 63, 67, 69]	Fabaceae	Kenjimbi	Fungal infections, tinea, (white spot, grille	INF/SKIN	L	H | R	T
MS 74/04	*Cassia alata* L [7, 8, 34, 37, 38, 42, 46, 49, 52, 59, 61, 63, 67, 69]	Fabaceae	Piaktie	Ggrille	SKIN	L	H	T
GW 71/04	*Cassia* sp.	Fabaceae	Pipi	Female infertility	REP	Root	D	O
MS 28/04	*Casuarina equisetifolia* L [33, 34, 38–40, 42, 43, 46, 58, 72]	Casuarinaceae	Kaiklee	Scabies, skin pox, small sores	SKIN	B	D	T
MS 19/04	*Cenchrus* sp.	Gramineae	Warawara	Cough	RESP	Stem	M	O
GW 94/04	*Cenchrus* sp.	Gramineae	Mitate	Enlarged spleen	ORG	L	D	O
GW 12/04	*Cerbera floribunda* K. Schum [51]	Apocynaceae	Yaung	Malaria, pneumonia	MAL/RESP	B	D	O
DK 20/05	*Cheilocostus speciosus* (J. König) C. Specht [8, 34, 42, 47, 56]	Costaceae	Yangir	Shortness of Breath	RESP	Succus	S	O
BK 008/06	*Christia* sp.	Fabaceae	Banjip	Diarrhoea, scabies, sores on the head like scabies	GAST/SKIN	L	D | R	O | T
MS 50/04	*Chrysopogon aciculatus* (Retz). Trin	Poaceae	Knarbru	Swollen bodies, legs, arms	SWELL	Whole	D	T
DK 54/05	*Cinnamonum* sp.	Lauraceae	Metamboi	Headache	HEAD	B	MS	T
GW 59/04	*Cissus* sp. [33, 34, 43, 53, 59, 62]	Vitaceae	Lenghasa	Stomach ache, diarrhoea	GAST	Sap	S	O
BK 049/06	*Clematis* sp. [8, 33, 34, 37, 39, 42, 43, 51, 53, 59, 65–67, 69]	Ranunculaceae	Gwawingga	Nasal congestion, running nose	RESP	L	V	I
GW 87/04	*Clerodendrum* sp. [8, 37, 38, 62]	Labiatae	Hambaihile	Snake bite	BITE	Sap	S	O
GW 91/04	*Clitoria ternatea* L.	Fabaceae	Pohuk	Determine female sex for baby, infertility	REP	Fruit	C	O
MS 78/04	*Cocos nucifera* L [7, 8, 34, 37–39, 43, 56, 59, 61, 67, 68]	Arecaceae	Niumour	Bleeding from cuts	WOUND	Fruit	H	T
BK 047/06	*Codiaeum variegatum* (L.) Rumph. ex A. Juss [8, 34, 35, 39, 42, 43, 48, 56, 58, 61, 62, 66, 73]	Euphorbiaceae	Diripmi	Ulcer	SKIN	Sap	S	T
MS 37/04	*Codiaeum variegatum* (L.) Rumph. ex A. Juss [8, 34, 35, 39, 42, 43, 48, 56, 58, 61, 62, 66, 73]	Euphorbiaceae	Waeke	Ssores around the mouth area	SKIN	Succus	S	T
DK 12/05	*Cordyline fruticosa* (L.) A. Chev [37, 38, 45, 56, 59–61]	Asparagaceae	Awa	Grille	SKIN	B & Stem	R	T
GW 86/04	*Cordyline fruticosa (L.) A. Chev* [37, 38, 45, 56, 59–61]	Asparagaceae	Haua	Fresh cuts, sores	WOUND/SKIN	L	H	T
MS 67/04	*Cordyline fruticosa (L.) A. Chev* [37, 38, 45, 56, 59–61]	Asparagaceae	Shir	Fever, headache, general body pain	FEV/HEAD/PAIN	L & yShoot	S	O
BK 053/06	*Crinum asiaticum* L [7, 8, 34, 39, 42, 49, 51, 56, 61, 62, 67, 74]	Amaryllidaceae	Yawal	Swollen leg, limbs, muscles	SWELL	L	H	T
GW 39/04	*Crinum asiaticum* L [7, 8, 34, 39, 42, 49, 51, 56, 61, 62, 67, 74]	Amaryllidaceae	Youri	General cleansing, swollen breast	GAST/SWELL	Sap & L	S | HR	O | T
MS 29/04	*Crinum asiaticum* L [7, 8, 34, 39, 42, 49, 51, 56, 61, 62, 67, 74]	Amaryllidaceae	Milakiap	Scabies, rectal prolapse	SKIN/GAST	Stem	S	O | T
MS 54/04	*Crinum asiaticum var. asiaticum* [34, 54, 61]	Amaryllidaceae	Kalava	Anemia	BLOOD	L	D	O
GW 75/04	*Cryptocarya* sp. [8, 33–35, 43, 50, 53, 55, 65]	Lauraceae	Misipi (misi-ph)	Cough, clear thinking	RESP/PSYCH	B	D	O
BK 035/06	*Cryptocarya* sp. [8, 33–35, 43, 50, 53, 55, 65]	Lauraceae	Kovi	Malaria and stomach ache	MAL/GAST	B	R	O
BK 029/06	*Curcuma longa* L [34, 42, 45]	Zingiberaceae	Laki	Poison by black magic	MAGIC	Root	R	O
GW 35/04	*Curcuma* sp. [34, 35, 40, 42–45, 62]	Zingiberaceae	Hivinguambe	Fever, headache	FEV/HEAD	Shoot	B	I
GW 38/04	*Curcuma* sp. [34, 35, 40, 42–45, 62]	Zingiberaceae	Lekienga	Broken bones, curds/boils	BONE/SKIN	L | Root	D	O
MS 84/04	*Cycas circinalis* L [34, 35, 38, 42, 43, 46, 47, 51, 73]	Cycadaceae	Malcoku/Malok	Sores	SKIN	Seed	R	T
BK 002/06	*Cycas rumphii* Miq [64]	Cycadaceae	Malehohong	Sores	SKIN	Seed	R	T
GW 90/04	*Cycas* sp. [34, 38, 39, 42, 43, 46, 47, 51, 63, 72, 73]	Cycadaceae	Rarier	Ulcers	SKIN	Seed	R	T
DK 06/05	*Cymbopogon citratus* (DC) Stapf [42, 56, 66]	Gramineae	Suimin	Fever	FEV	Whole	V	T
GW 53/04	*Cymbopogon citratus* (DC) Stapf [42, 56, 66]	Gramineae	Yamawi	Malaria	MAL	L	V	I
MS 70/04	*Davallia* sp. [8, 34]	Davalliaceae	Klakol	Headache, fever	HEAD/FEV	Sap	C	O
DK 35/05	*Dendrocnide cordata* (Warb. ex H.J.P. Winkl.) Chew [51]	Urticaceae	Chumbia	Body aches	PAIN	L	R	T
MS 33/04	*Dendrocnide latifolia* (Gaudich.) Chew [64]	Urticaceae	Shalat (green)	General body pain	PAIN	L	R	T
GW 101/04	*Desmodium* sp. [7, 9, 33–35, 37, 43, 48, 60, 66, 68, 70, 72, 75, 76]	Fabaceae	Ninji	Contraceptive	REP	Root	S	O
MS 81/04	*Dillenia* sp. [39, 50, 58, 60, 65, 77]	Dilleniaceae	Kol	Fever, headache, cough	FEV/HEAD/RESP	L	S	O
DK 59/05	*Dioscorea bulbifera* L [33, 42]	Dioscoreaceae	Remsik	Contraceptive	REP	Seed	S	O
GW 63/04	*Dioscorea* sp. [7, 8, 33, 35, 38, 42, 43, 46, 54, 59, 72, 73]	Dioscoreaceae	Harehare	Headache, migraine	HEAD	L	HR	T
BK 013/06	*Donax canniformis* (G. Forst.) K. Schum [8]	Marantaceae	Gani	Ear ache	PAIN	yL	R	T
DK 23/05	*Donax canniformis* (G. Forst.) K. Schum [8]	Marantaceae	Guarimb	Ear infection	INF	L	R	T
GW 78/04	*Dracaena angustifolia* (Medik.) Roxb [42, 58]	Asparagaceae	Hembesaihe	Fever, headache, stomach complaints	FEV/HEAD/GAST	Root	S	O
GW 25/04	*Dysoxylum* sp. [33, 34, 39, 41, 51, 59, 63]	Meliaceae	Sengiwama	Sores, ulcers	SKIN	B	R	T
GW 68/04	*Dysoxylum* sp. [33, 34, 39, 41, 51, 59, 63]	Meliaceae	Huambuka	Malaria, cough	MAL/RESP	L	D	O
GW 100/04	*Elaeocarpus sphaericus* Schum [39, 53]	Elaeocarpaceae	Nangila	Malaria, cough, pneumonia, shortness of breath	MAL/RESP	B	D	O
MS 25/04	*Elaeocarpus sphaericus* Schum [39, 53]	Elaeocarpaceae	Kaiboun	Asthma	RESP	B	S	O
BK 043/06	*Elatostema* sp [8, 33–35, 39–41, 43, 53, 65, 66, 69]	Urticaceae	Kaskas-bhirs	Scabies	SKIN	Whole	D	T
MS 59/04	*Elatostema* sp [8, 33–35, 39–41, 43, 53, 65, 66, 69]	Urticaceae	Moin kukuri	Fever, headache, joint pain, fertility	FEV/HEAD/PAIN/REP	Whole	M	O
GW 28/04	*Endospermum formicarium* Becc [7, 34, 39, 43, 62, 67]	Euphorbiaceae	Bundua	Fever, asthma	FEV/RESP	B	S	O
DK 40/05	*Endospermum labios* Schodde [7, 34, 39, 43, 62, 67]	Euphorbiaceae	Paruang	Scabies	SKIN	Seed & Flower	C	O
MS 89/04	*Endospermum medullosum* L.S.Sm.	Euphorbiaceae	Kakar	Fever, body pain, unconscious	FEV/PAIN/PSYCH	L	B	I
GW 47/04	*Epipremnum pinnatum* (L.) Engl [7, 8, 34, 39, 42, 67]	Araceae	Kumbui-bhi	Fever	FEV	B	S	O
BK 009/06	*Epipremnum* sp. [7, 8, 34, 39, 42, 43, 66, 67]	Araceae	Kunga	Dysentery (excreting of blood), vomiting of blood	GAST	Root	M | C	O
MS 12/04	*Epipremnum* sp. [7, 8, 34, 39, 42, 43, 66, 67]	Araceae	Klakial	Headache, swollen bodies, fever, cold	HEAD/SWELL/FEV/RESP	Sap	S	O
GW 18/04	*Erythrina merrilliana* Krukoff	Fabaceae	Kwai	Diarrhoea, shortness of breath,cough	GAST/RESP	L & B	D	O
MS 42/04	*Erythrina merrilliana* Krukoff	Fabaceae	Pear	Contraceptive	REP	B	B	O
MS 52/04	*Euodia hortensis* J.R. Forst. & G. Forst. [8, 34, 42, 43, 53, 56, 57, 63]	Rutaceae	Ghin	Unconsciousness	PSYCH	L	V	I
MS 66/04	*Euodia* sp. [8, 33, 34, 40, 42, 43, 53, 56, 57, 62, 63]	Rutaceae	Muth	Fertility,emetic	REP/GAST	B	S	O
BK 025/06	*Euphorbia heterophylla* L [51]	Euphorbiaceae	Wilai	For treating diarrhoea	GAST	Sap	S	O
BK 023/06	*Euphorbia hirta* L [9, 34, 39, 46, 50, 51, 56, 57, 67]	Euphorbiaceae	Unknown	Sore	SKIN	L	S	T
GW 17/04	*Euphorbia hirta* L [9, 34, 39, 46, 50, 51, 56, 57, 67]	Euphorbiaceae	Seplein Nai	Shortness of breath, asthma, pneumonia	RESP	Whole	D	O
DK 03/05	*Euphorbia plumerioides* Teijsm. ex Hassk. [33, 34, 36, 43, 51, 53, 60, 69]	Euphorbiaceae	Miambi/Pombi	Poisoning	POIS	Sap	S	O
MS 47/04	*Euphorbia* sp. [9, 34–36, 41–44, 50, 53, 54, 57–59, 67, 74, 78]	Euphorbiaceae	Sungwia	Emetic	GAST	Sap	S	O
GW 44/04	*Euphorbia* sp. [9, 34–36, 41–44, 50, 53, 54, 57–59, 67, 74, 78]	Euphorbiaceae	Wale	Emetic	GAST	Sap	S	O
GW 80/04	*Euphorbia* sp. [9, 34–36, 41–44, 50, 53, 54, 57–59, 67, 74, 78]	Euphorbiaceae	Tuth	Emetic	GAST	Sap	S	O
MS 79/04	*Euphorbia tithymaloides* (L.) [51, 56]	Euphorbiaceae	Mual nias	Epigastric pain	GAST	Sap	S	O
BK 046/06	*Ficus adenosperma* Miq [8, 33–35, 53]	Moraceae	Belloki	Cut	WOUND	yL	S	T
DK 41/05	*Ficus pungens* Reinw. ex Blume [9, 33, 34, 42, 43, 53, 59, 60, 63]	Moraceae	Kuar	Shortness of breath	RESP	Succus	S	O
MS 40/04	*Ficus septica* Burm.f. [34, 37–40, 42, 43, 48, 49, 51, 57–59, 61, 62, 66, 67, 78]	Moraceae	Poipuk	Diarrhoea	GAST	Sap & yShoot	S	O
DK 58/05	*Ficus* sp. [7–9, 33–40, 42–45, 47–49, 51–59, 61–63, 66–69, 74, 75, 77, 78]	Moraceae	Tuohepolehe	Malnutrition	NUT	Sap	C	O
GW 58/04	*Ficus* sp. [7–9, 33–40, 42–45, 47–49, 51–59, 61–63, 66–69, 74, 75, 77, 78]	Moraceae	Manjemieri	Nutrient supplement for babies	NUT	Sap	S	O
GW 74/04	*Ficus* sp. [7–9, 33–40, 42–45, 47–49, 51–59, 61–63, 66–69, 74, 75, 77, 78]	Moraceae	Wavihasa/Horikieng	Broken bones	BONE	Root	M	T
GW 89/04	*Ficus* sp. [7–9, 33–40, 42–45, 47–49, 51–59, 61–63, 66–69, 74, 75, 77, 78]	Moraceae	Chiplapul	Abortion	REP	B	R	T
MS 17/04	*Ficus* sp. [7–9, 33–40, 42–45, 47–49, 51–59, 61–63, 66–69, 74, 75, 77, 78]	Moraceae	Bukabok	Fracture	BONE	B	R	T
MS 31/04	*Ficus* sp. [7–9, 33–40, 42–45, 47–49, 51–59, 61–63, 66–69, 74, 75, 77, 78]	Moraceae	Moul koni	Ulcer	SKIN	Sap	S	T
MS 88/04	*Ficus* sp. [7–9, 33–40, 42–45, 47–49, 51–59, 61–63, 66–69, 74, 75, 77, 78]	Moraceae	Aiyau	Toothache	DENT	yRoot	M	O
MS 75/04	*Ficus wassa* Roxb [33, 34, 39, 40, 42, 47, 68, 75]	Moraceae	Kikquai	Contraceptive	REP	Root	M	O
BK 060/06	*Gnetum gnemon* L [8, 34]	Gnetaceae	Yit	Removal of wood or stick in skin	WOUND	yL	S	T
DK 14/05	*Gnetum gnemon* L [8, 34]	Gnetaceae	Mogsa	Removal of nails/ splints lodged in the body	WOUND	Sap	S	T
MS 18/04	*Gnetum gnemon* L [8, 34]	Gnetaceae	Popoyiri	Eye disease	OCC	Sap	S	T
GW 45/04	*Gnetum gnemonoides* Brongn.	Gnetaceae	Biek	Fever, headache (malaria)	FEV/MAL	B	D	O
MS 14/04	*Graptophyllum* sp. [7–9, 33, 35, 36, 39, 41, 66, 67]	Acanthaceae	Inta’niat	Fever, headache, joint pain, cold	FEV/HEAD/ PAIN/RESP	L	D	O & I & T
GW 11/04	*Gymnostoma papuanum* (S. Moore) L.A.S. Johnson [33, 35, 43]	Casuarinaceae	Mania	Shortness of breath, asthma	RESP	B	D	O
GW 70/04	*Hemigraphis reptans* (G. Forst.) T. Anderson ex Hemsl.	Acanthaceae	Mijika	Centipede bite	BITE	Whole	HR	T
BK 018/06	*Hibiscus rosa-sinensis* L [37, 56, 59]	Malvaceae	Mawe	Sore eye	OCC	Flower	R	T
DK 13/05	*Hibiscus rosa-sinensis* L [37, 56, 59]	Malvaceae	Kupawaruk	Menstrual cramps	REP	L	S	O
MS 05/04	*Homalanthus* sp. [7, 8, 33–36, 42, 50, 51, 58]	Euphorbiaceae	War moap	Scabies	SKIN	Stem	D	T
DK 42/05	*Homalium foetidum* (Roxb.) Benth [8]	Salicaceae	Mes	Knee ache	PAIN	B	MAG	P_to_Plant
GW 83/04	*Hydriastele costata* F.M. Bailey	Arecaceae	Yawah	Shortness of breath	RESP	Stem	S	O
DK 33/05	*Intsia bijuga* (Colebr.) Kuntze	Fabaceae	Wun	Boil	SKIN	Stem	C	T
GW 08/04	*Intsia bijuga* (Colebr.) Kuntze	Fabaceae	Hwapo	Fractured bones	BONE	B	HR	T
MS 46/04	*Intsia bijuga* (Colebr.) Kuntze	Fabaceae	Tou’r	Severe back pain	PAIN	B	D	O & T
GW 52/04	*Ipomea* sp. [35, 36, 39, 43, 49, 61]	Convolvulaceae	Firac	Distended stomach, pigbel	GAST	L	C	O
BK 020/06	*Ipomoea pes-caprae* (L.) R. Br [59]	Convolvulaceae	Waimabhu	Running nose, cough, asthma	RESP	Stem	S	?
MS 26/04	*Ipomoea pes-caprae* (L.) R. Br [59]	Convolvulaceae	Kairo	Fever, headache, joint pain, swelling of the body	FEV/HEAD/PAIN/SWELL	L	S	O
BK 021/06	*Kalanchoe pinnata* (Lam.) Pers [34, 39, 46, 51, 67]	Crassulaceae	Kulukir	knee pain/ache, back ache/pain, swollen legs, boils	PAIN/ SWELL /SKIN	L	H	T
DK 04/05	*Kalanchoe pinnata* (Lam.) Pers [34, 39, 46, 51, 67]	Crassulaceae	Asamambia	Insect bite	BITE	L	H	T
BK 015/06	*Laportea decumana* Wedd. [9, 32, 34–37, 39, 42–45, 49, 51, 53, 59, 63, 65, 66, 69, 71, 72, 74–77]	Urticaceae	Salat	Muscle ache, knee pain, ankle sprain	PAIN	L	R	T
DK 32/05	*Laportea interrupta* (L.) Chew [34, 39, 51, 67, 73]	Urticaceae	Shalat (red)	Fresh cuts,wounds	WOUND	yL	H	T
BK 027/06	*Leucosyke capitellata* Wedd [8, 9]	Urticaceae	Elan	Sores and cuts	SKIN/WOUND	B	R	T
GW 22/04	*Litsea* sp. [8, 9, 33, 43, 69]	Lauraceae	Erikombi	Cough, malaria	RESP/MAL	L	D	O
GW 23/04	*Litsea* sp. [8, 9, 33, 43, 69]	Lauraceae	Neimie	Malaria, fevers, coughs	MAL/FEV/RESP	L or B	D	O
GW 06/04	*Macaranga clavata* Warb.	Euphorbiaceae	Lambie	Skin infections, scabies	SKIN	B	S	T
MS 11/04	*Macaranga darbyshirei* Airy Shaw	Euphorbiaceae	Walmieng	Anti-venom	POIS	B	M	O
GW 46/04	*Maclura cochinchinensis* (Lour.) Corner [39]	Moraceae	Lomowi	Cough, stomach complaints	RESP/GAST	Stem	S	O
GW 93/04	*Mangifera indica* L [8, 34, 51, 56]	Anacardiaceae	Huarambie/Wamahang	Snake bite	BITE	B	D | H	O & T
DK 51/05	*Manihot esculenta* Crantz [34, 35, 51]	Euphorbiaceae	Gumbyow	Fresh cut,wounds	WOUND	Root	R	T
MS 16/04	*Marattia* sp. [7, 33, 34, 36, 43, 54, 69]	Marattiaceae	Rireo	Fever, headache, swollen bodies etc.	FEV/HEAD/SWELL/OTHER	yShoot	S	O
BK 011/06	*Melanolepis multiglandulosa* (Reinw. ex Blume) Rchb. & Zoll [34]	Euphorbiaceae	Wamakhir	Snake bites	BITE	B	M	O
DK 36/05	*Melanolepis multiglandulosa* (Reinw. ex Blume) Rchb. & Zoll [34]	Euphorbiaceae	Waru	Snake bite	BITE	B	M	O
GW 02/04	*Melanolepis multiglandulosa* (Reinw. ex Blume) Rchb. & Zoll [34]	Euphorbiaceae	Warimaing	Snake and centipede bites, antivenom	BITE/POIS	B	M	O
MS 36/04	*Melastoma* sp. [7–9, 33, 34, 43, 47, 54]	Melastomataceae	Mutamuth	Blocked nose, flu, cough	RESP	L	V	I
DK 05/05	*Melicope triphylla* (Lam.) Merr [34, 40, 42, 43, 62]	Rutaceae	Kupun	Abortion	REP	L	D	O
BK 001/06	*Merremia peltata* (L.) Merr [8, 34, 42, 43, 56, 59, 67]	Convolvulaceae	Aukut	Boil, sore or ulcer, fresh cut	SKIN/WOUND	Sap | L	S | H	T
DK 28/05	*Merremia peltata* (L.) Merr [8, 34, 42, 43, 56, 59, 67]	Convolvulaceae	Bangpuk	Fresh cuts, /wounds	WOUND	Sap	S	T
GW 62/04	*Merremia peltata* (L.) Merr [8, 34, 42, 43, 56, 59, 67]	Convolvulaceae	Nangumareng	Determine male sex of baby	REP	L	D	O
GW 43/04	*Merremia* sp. [8, 34, 42, 43, 56, 59, 67]	Convolvulaceae	Wararamang	Fever, malaria	FEV/MAL	Stem	S	O
DK 30/05	*Metroxylon sagu* Rottb [42]	Arecaceae	Nouk	Burns	BURN	Stem	R	T
GW 96/04	*Mikania* sp.	Asteraceae	Lihasuanga	Skin infections, scabies, sores	SKIN	Sap	S	T
BK 059/06	*Mimosa pudica* L [8, 34]	Fabaceae	Bambu kiya	Induce sleep	PSYCH	Whole	D	T
DK 52/05	*Mimosa pudica* L [8, 34]	Fabaceae	Haihiksa	Infant colic	CHILD	Whole	D	T
MS 77/04	*Mimosa pudica* L [8, 34]	Fabaceae	Miatmiat	Induced sleep	PSYCH	Whole	D	T
GW 20/04	*Mitracarpus* sp.	Rubiaceae	Waramang	Eye infections, color defects	OCC	Whole	B	I
BK 038/06	*Morinda citrifolia* L [7, 8, 34, 37, 38, 42, 43, 48, 49, 56–59, 61, 68, 70, 73, 78]	Rubiaceae	Simbiya	Knee ache, cough	PAIN/RESP	yL | Fruit	D | R or H	O & T
MS 71/04	*Morinda citrifolia* L [7, 8, 34, 37, 38, 42, 43, 48, 49, 56–59, 61, 68, 70, 73, 78]	Rubiaceae	Knuel	General body pain, boils, inflammation	PAIN/SKIN/SWELL	L	R	T
GW 64/04	*Mucuna novo-guineensis* Scheff. [8]	Fabaceae	Kilemiesik	Shortness of breath	RESP	Root	S	O
MS 37/04	*Mucuna* sp. [8, 9, 34, 43, 47, 50, 52, 56, 63]	Fabaceae	Ombo	Anemia	BLOOD	Sap	S	O
GW 51/04	*Mucuna* sp. [8, 9, 34, 43, 47, 50, 52, 56, 63]	Fabaceae	Wamayihara	Tooth ache, loose tooth	DENT	Stem	M	O
GW 66/04	*Mucuna* sp. [8, 9, 34, 43, 47, 50, 52, 56, 63]	Fabaceae	Ponambile	Anemia	BLOOD	B	S	O
GW 84/04	*Mucuna* sp. [8, 9, 34, 43, 47, 50, 52, 56, 63]	Fabaceae	Manvil	Arthritis joint pain, back ache	PAIN	B	S	T
BK 003/06	*Murraya paniculata* (L.) Jack [8, 73]	Rutaceae	Sika	Ccough	RESP	L	D	O
DK 24/05	*Musa acuminata* Colla [34, 37, 47, 57]	Musaceae	Yup	Sore lip	PAIN	Fruit	C	T
MS 44/04	Musa sp. [8, 9, 32–34, 37, 42, 43, 47, 52, 54, 56, 57, 59, 61, 63, 67, 71, 72]	Musaceae	Wur karasau	Wound	WOUND	Sap	S	T
BK 055/06	*Nauclea orientalis* (L.) L [34, 55, 63]	Rubiaceae	Runggool	Asthma,shortness of breath	RESP	B	S	O
DK 44/05	*Nauclea orientalis* (L.) L [34, 55, 63]	Rubiaceae	Kuva	Snake bite	BITE	B	S	O
GW 10/04	*Neonauclea purpurea* (Roxb.) Merr [39]	Rubiaceae	Kripa	Fever, headache (malaria), pneumonia, asthma	FEV/MAL/RESP	B	B	I & O
BK 061/06	*Neonauclea* sp.	Rubiaceae	Gipma	Poisonous snake bite	BITE	B	M	O
DK 48/05	*Nephrolepis hirsutula* (G. Forst.) C. Presl [8]	Lomariopsidaceae	Tamanguia	Uncontrollable urine	URINE	L	C	O
GW 36/04	*Nephrolepis* sp. [7, 8, 33, 34, 43]	Lomariopsidaceae	Walendau	Headache, fever (malaria)	HEAD/MAL	Shoot & Root	S	O
MS 48/04	*Nicotiana* sp. [33–36, 42, 43, 47, 52, 59, 63, 66, 71, 72, 76, 77]	Solanaceae	Kennings	Anticoagulant	BLOOD	yL	H	T
BK 024/06	*Nicotiana tabacum* (L.) [33, 35, 36, 42, 43, 52, 59, 63, 76, 77]	Solanaceae	Saukien	Sores	SKIN	L	S	T
BK 036/06	Not Identified	Not Identified	Ukapuk	Scabies, malaria	SKIN/MAL	Sap	S	T | O
DK 47/05	Not identified	Not identified	Kupnenj	Shortness of breath	RESP	Succus	S	O
DK 60/05	Not identified	Fabaceae	Wulamian	Malnutrition	NUT	Whole	H	O
MS 80/04	Not identified	Orchidaceae	Kraufung	Skin disease (grille)	SKIN	L	H	T
DK 57/05	Not identified	Not identified	Sarimbiya	Cough	RESP	L	-	O
MS 22/04	Not identified	Not identified	Asakurkunja	Scabies	SKIN	Stem & Root	D	T
MS 08/04	*Ocimum basilicum* L [32, 34, 38, 39, 42, 43, 46, 48, 65, 67, 73]	Labiate	Ruk	General body weakness, fever, headache, etc.	FEV/MAL/HEAD	Whole	B	I
BK 004/06	*Octomeles sumatrana* Miq [43]	Datiscaceae	Wani	Asthma, back ache, malnourished/pigbel	RESP/PAIN/NUT/GAST	B | B | Sap	S	O | O | O
GW 48/04	*Octomeles sumatrana* Miq [43]	Datiscaceae	Waine	Fever	FEV	B	S	O
MS 30/04	*Pandanus dubius* Spreng.	Pandanaceae	Viak	Asthma	RESP	yShoot	S	O
GW 98/04	*Pangium edule* Reinw [34, 42, 43, 50, 51, 53]	Achariaceae	Imahek	Enlarged spleen	ORG	Fruit	R	O
MS 35/04	*Pangium edule* Reinw [34, 42, 43, 50, 51, 53]	Achariaceae	Sis	Lice killer	INSECTICIDE	L	S	T
GW 65/04	*Papuechites* sp. [34, 43]	Apocynaceae	Pari	Enlarged spleen	ORG	Fruit	S	O
GW 29/04	*Parsonia* sp. [57, 59]	Apocynaceae	Tielimbika	Fresh cuts, sores	SKIN/WOUND	L	H	T
BK 032/06	*Passiflora foetida* L [8, 42, 51, 56]	Passifloraceae	Bombo	Asthma, white spot	RESP/SKIN	Flower & L | Seed	D | R	O & T
DK 46/05	*Passiflora foetida* L [8, 42, 51, 56]	Passifloraceae	Apsarapuk	Whitespots	SKIN	L	R	T
GW 19/04	*Passiflora foetida* L [8, 42, 51, 56]	Passifloraceae	Apduanpuk	Strong cough	RESP	Shoot & L	S	O
MS 09/04	*Passiflora foetida* L [8, 42, 51, 56]	Passifloraceae	Maparou	Skin disease	SKIN		R	T
DK 55/05	*Passiflora* sp. [8, 42, 51, 56]	Passifloraceae	War yasokk	Scabies	SKIN	Sap	H	T
BK 017/06	*Peperomia pellucida* (L.) Kunth [7]	Piperaceae	Koikoiwara	Pimple	SKIN	L	R	T
GW 81/04	*Peperomia pellucida* (L.) Kunth [7]	Piperaceae	Lerek	Fever, headache, (malaria)	FEV/MAL	Whole	D	O
MS 58/04	*Peperomia pellucida* (L.) Kunth [7]	Piperaceae	Kinkanak	Antidepressant	PSYCH	L	D	T
BK 014/06	*Phrynium* sp. [45]	Marantaceae	Ripa kwalingu	Scabies	SKIN	Succus	S	T
MS 55/04	*Phyllanthus amarus* Schumach. & Thonn [34, 43, 49, 52, 63]	Phyllanthaceae	Kambaningi	Fever, headache, swollen bodies	FEV/HEAD/SWELL	Root	S	O
GW 54/04	*Phyllanthus niruri* L [7, 9, 34, 42, 43, 46, 57, 59, 73]	Phyllanthaceae	Hipanchinchi	Menorrhagia	REP	Whole	D	O
MS 60/04	*Phyllanthus niruri* L [7, 9, 34, 42, 43, 46, 57, 59, 73]	Phyllanthaceae	Shuk miau	Fever	FEV	Whole	D	T
GW 14/04	*Phyllanthus* sp. [7, 9, 33–35, 39, 42, 43, 46, 49, 52, 57, 59, 63, 67, 73]	Phyllanthaceae	Kai veai	Tooth infections, toothache	DENT	Root	M	T
GW 61/04	*Pimelodendron amboinicum* Hassk [34, 39]	Euphorbiaceae	Sombik	Enlarged spleen	ORG	Sap	S	O
MS 15/04	*Pimelodendron amboinicum* Hassk [34, 39]	Euphorbiaceae	Kunial	Swollen stomach	GAST	B	D	T
BK 062/06	*Piper betle* L [37–39, 42, 61, 67, 68]	Piperaceae	Kwashe gungga	Sores,boils	SKIN	L	H	T
DK 22/05	*Piper betle* L [37–39, 42, 61, 67, 68]	Piperaceae	Kosh	Abdominal ache	GAST	Seed	MS	T
GW 49/04	*Piper betle* L [37–39, 42, 61, 67, 68]	Piperaceae	Guspui	Tuberculosis, centipede bite	BITE/INF	L | Fruit	H	O | T
DK 27/05	*Piper mestonii* F.M. Bailey.	Piperaceae	Hrunga	Fresh cuts, wounds	WOUND	L	S	T
GW 97/04	*Piper* sp. [8, 9, 33, 34, 37–39, 42, 43, 53, 55, 57, 58, 60, 61, 65, 67, 68, 70, 74]	Piperaceae	Walehru	Memory enhancing, clear thinking	PSYCH	Root	M	O
MS 56/04	*Piper* sp. [8, 9, 33, 34, 37–39, 42, 43, 53, 55, 57, 58, 60, 61, 65, 67, 68, 70, 74]	Piperaceae	Kunek	Anesthetic	PAIN	Root	S	O
DK 31/05	*Piscidia grandifolia* (Donn. Sm.) I.M. Johnst. [8]	Fabaceae	Yinapuk	Strong headache	HEAD	Stem	R	T
GW 32/04	*Pisonia longirostris* Teijsm. & Binn [56]	Nyctaginaceae	Kumie/Weworo	Tropical ulcers, peptic ulcers	SKIN/GAST	B	S	T | O
DK 45/05	*Planchonia papuana* R. Knuth	Lecythidaceae	Ningia	Scabies	SKIN	B	C	O
MS 57/04	*Plectranthus amboinicus* (Lour.) Spreng [60, 74]	Labiatae	Wasirika	Skin disease (grille)	SKIN	L	S	T
GW 13/04	*Plectranthus hereroensis* Engl.	Labiatae	Sumoun	Stomach ulcers, placenta sores	GAST/REP	L	D	O
GW 15/04	*Plectranthus hereroensis* Engl.	Labiatae	Krau sumin	Scabies, itchy skin	SKIN	L	S	T
BK 031/06	*Plectranthus parviflorus* Willd.	Labiatae	Humbiang	Sores	SKIN	L	S	T
MS 49/04	*Plectranthus scutellarioides* (L.) R.Br [8, 9, 33, 42, 43, 56, 58, 74]	Labiatae	Humbiang	Ulcer, fresh cut	SKIN/WOUND	L	S	T
MS 87/04	*Plectranthus scutellarioides*(L.) R.BR [8, 9, 33, 42, 43, 56, 58, 74]	Labiatae	Trakain	Skin disease (grille)	SKIN	L	R	T
GW 30/04	*Pongamia pinnata* (L.) Pierre [40, 42, 43, 52, 56, 59, 63]	Fabaceae	Lai	Skin infections, scabies	SKIN	Root	S	T
GW 41/04	*Pouteria* sp.	Sapotaceae	Pokware	Scabies, grille	SKIN	Sap	S	T
BK 052/06	*Premna serratifolia* L. [39, 42, 61]	Lamiaceae	Kunggwia	Emetic	GAST	Seed	R	O
GW 42/04	*Premna* sp.[8, 34, 39, 42, 43, 53, 56, 63]	Lamiaceae	Ningrik	Ear ache	PAIN	B	S	T
MS 06/04	*Premna* sp.[8, 34, 39, 42, 43, 53, 56, 63]	Lamiaceae	Wurweik	Malaria	MAL	L & B	D	O
BK 050/06	*Psidium guajava* L [39, 40, 56, 61]	Myrtaceae	Yambu	Cough, itchy throat	RESP	Fruit	R	O
DK 17/05	*Psidium guajava* L [39, 40, 56, 61]	Myrtaceae	Yambosik	Diarrhoea	GAST	L	D	O
DK 49/05	*Psychotria* sp. [7, 9, 33–35, 37, 38, 43, 59]	Rubiaceae	Sisikupa	Boil	SKIN	L	S	T
GW 07/04	*Psychotria* sp. [7, 9, 33–35, 37, 38, 43, 59]	Rubiaceae	Konumbo	Enlarged spleen	ORG	Sap	S	O
BK 040/06	*Pterocarpus indicus* Willd. [7, 8, 34, 37, 38, 40, 42, 43, 47, 48, 56, 57, 59, 61, 67, 68]	Fabaceae	Markulu	Anemia	BLOOD	Sap	S	O
GW 03/04	*Pterocarpus indicus* Willd. [7, 8, 34, 37, 38, 40, 42, 43, 47, 48, 56, 57, 59, 61, 67, 68]	Fabaceae	Moroho	Diarrhoea, stomach ache, anemia	GAST/BLOOD	L & B	D | D	O
DK 18/05	*Riedelia corallina* (K. Schum.) Valeton	Zingiberaceae	Moukuaikuai	Menstrual cramps	REP	Root	D	O
MS 63/04	*Scaevola sericea* Vahl [39]	Goodeniaceae	Knanas	Cough	RESP	yL	S	O
MS 83/04	*Schismatoglottis calyptrata* (Roxb.) Zoll. & Moritzi	Araceae	Maghau	Sore	SKIN	L	H	T
GW 55/04	*Semecarpus* sp. [8, 50, 51]	Anacardiaceae	Huaho	Itchy skin (pruritis)	SKIN	B	D	T
MS 76/04	*Sida rhombifolia* L [7, 8, 34, 37, 40, 42, 43, 50, 51, 54, 57, 59, 60, 73]	Malvaceae	Shasar	Contraceptive	REP	Root	M	O
GW 99/04	*Smilax* sp.	Smilacaceae	Kilembole	Generalcleansing	MAINT	Root & Stem	S	O
GW 26/04	*Solanum torvum* Sw [8, 34, 42, 48, 68]	Solanaceae	Warandangu/Waramande	Joint pains,arthritis	PAIN	Root	D	O
GW 33/04	*Spathiphyllum* sp.	Araceae	Hwembung	Strong cough, fever	RESP/FEV	Root	S	O
GW 95/04	*Sphaerostephanos* sp. [7, 8, 33, 42, 53]	Thelypteridaceae	Ningi	Malaria	MAL	Root	D	O
MS 24/04	*Sphaerostephanos unitus* (L.) Holttum [33, 53]	Thelypteridaceae	Kipokip	Sores, ulcers	SKIN	L	S	T
DK 50/05	*Spondias dulcis* Parkinson [43]	Anacardiaceae	Nungwi	Scabies	SKIN	B	C	O
GW 67/04	*Spondias dulcis* Parkinson [43]	Anacardiaceae	Akanang	Sores, scabies	SKIN	Shoot	S	O
GW 37/04	*Stephania japonica* var. discolor (Blume) Forman [51]	Menispermaceae	Poponga	Malaria	MAL	Root	S	O
GW 31/04	Stephania sp. [34, 37, 38, 43, 47, 48, 51]	Menispermaceae	Yuamareng/Kenduek	Fever, headache (malaria), asthma, cough	FEV/MAL/RESP	Sap	S	O
GW 04/04	*Sterculia shillinglawii* F. Muell.	Malvaceae	Huasiva or Chosembi	Enlarged spleen, pigbel	ORG/GAST	L | Sap	D | S	O
DK 09/05	*Syzygium malaccense* (L.) Merr. & L.M. Perry [7, 8, 34, 39, 42, 47, 56, 67]	Myrtaceae	Gwangolik	Fever	FEV	L	D	T
DK 10/05	*Syzygium malaccense* (L.) Merr. & L.M. Perry [7, 8, 34, 39, 42, 47, 56, 67]	Myrtaceae	Turukirmba	Fever	FEV	L	D	T
MS 43/04	*Syzygium* malaccense (L.) Merr. & L.M. Perry [7, 8, 34, 39, 42, 47, 56, 67]	Myrtaceae	Duokuma	Epigastric pain	GAST	L	H	T
BK 048/06	*Syzygium* sp. [8, 9, 33, 43, 45]	Myrtaceae	Kaviak	Cough with itchy throat	RESP	yL	D	O
BK 054/06	*Tabernaemontana pandacaqui* Lam [34, 38, 46]	Apocynaceae	Karaban	Grille	SKIN	Fruit	S	T
GW 76/04	*Tabernaemontana* sp.	Apocynaceae	Raviapari	Determine baby girl	REP	Root	M	O
GW 82/04	*Tinospora arfakiana* Becc.	Menispermaceae	Saihuna	Cough, grille	RESP/SKIN	L	D | S	O | T
MS 65/04	*Tinospora* sp.	Menispermaceae	Tifoniak kuriri	Asthma, cough	RESP	L	S	O
GW 57/04	*Tylophora* sp.	Asclepiadaceae	Yousa	Recovery from illness	NUT	Root	D	O
BK 005/06	*Uncaria lanosa* var. appendiculata (Benth.) Ridsdale	Rubiaceae	Marangi	Fever, headache, malaria, cough, malnutrition	MAL/FEV/NUT/HEAD/RESP	Sap	S	O
MS 82/04	*Uncaria lanosa* var. appendiculata (Benth.) Ridsdale	Rubiaceae	Mewow	Severe fever, chronic diarrhoea with blood, loss of weight.	FEV/GAST	Sap	S	O
DK 29/05	*Uncaria orientalis* Guillaumin	Rubiaceae	Marange	Shortness of breath	RESP	Sap	S	O
GW 85/04	*Uncaria* sp. [8, 9, 33, 35, 39]	Rubiaceae	Trakiau kakoin	Headache, migraine	HEAD	Sap	S	O
GW 72/04	Ursi sp.	Fabaceae	Swamareng	Determine baby boy	REP	Root	S	O
GW 77/04	*Urticastrum decumanum* (Roxb.) Kuntze [9, 32, 34–37, 39, 42–45, 49, 51, 53, 59, 63, 65, 66,69, 71, 72, 74–77]	Urticaceae	Purkumb	B body, muscle, joint pains, pneumonia	PAIN/RESP	L	R	O | T
MS 62/04	*Urticastrum decumanum (Roxb.) Kuntze* [9, 32, 34–37, 39, 42–45, 49, 51, 53, 59, 63, 65, 66 69, 71, 72, 74–77]	Urticaceae	Chipia	Abortion	REP	L	D	O
BK 012/06	*Vanilla* sp. [65]	Orchidaceae	Dunauru banguwi	Prevent miscarriage	REP	Sap	S	O
MS 13/04	*Villebrunea* sp.	Urticaceae	Wurarian	Very high fever, headache, swollen bodies	FEV/HEAD/SWELL	Sap	S	O
MS 86/04	*Virola surinamensis* (Rol. ex Rottb.) Warb [33]	Myristicaceae	Sukuai	Sore in the baby’s mouth	CHILD	L	MS	T
GW 73/04	*Wedelia biflora* (L.) DC. [34, 38, 39, 42, 43, 46, 48, 52, 56, 57, 59, 62, 63, 67, 68, 70]	Asteraceae	Bambawhoo	Cough, diarrhoea, women’s bleeding disorders	RESP/GAST/REP	L	D	O
BK 019/06	*Wedelia* sp.[34, 38, 39, 42, 43, 46, 48, 52, 56, 57, 59, 62, 63, 67, 68, 70]	Asteraceae	Pava	Running nose, cough,asthma	RESP	L	V	I
MS 72/04	*Wedelia* sp.[34, 38, 39, 42, 43, 46, 48, 52, 56, 57, 59, 62, 63, 67, 68, 70]	Asteraceae	Kiskiash	Toothache	DENT	yShoot	M	O
BK 030/06	*Zingiber officinale* Roscoe [8, 9, 34, 36, 39, 42, 53, 56, 60, 63, 65–67, 71, 73, 77]	Zingiberaceae	Kambei laki	Ssnake bites	BITE	L	R	O & T
DK 07/05	*Zingiber officinale* Roscoe [8, 9, 34, 36, 39, 42, 53, 56, 60, 63, 65–67, 71, 73, 77]	Zingiberaceae	Nikirkuasa	Malaria	MAL	Whole	D	T
DK 39/05	*Zingiber officinale* Roscoe [8, 9, 34, 36, 39, 42, 53, 56, 60, 63, 65–67, 71, 73, 77]	Zingiberaceae	Huaukuasa	Malaria	MAL	Whole	D	O
MS 45/04	*Zingiber officinale* Roscoe [8, 9, 34, 36, 39, 42, 53, 56, 60, 63, 65–67, 71, 73, 77]	Zingiberaceae	Leai	Epigastric pain, vomiting, diarrhoea	GAST	Root	MS	O & T

Ailment treated (Ailmentcode) as follows: BITE = insect or snake bite; BLOOD = hematological issues including coagulation; BONE = bone related injury or disease; BURN = burns; CANC = cancer; CHILD = childhood disease; CV = Cardiovascular; DENT = dental disease; FEV = fever; GAST = gasteroenterological disease; HEAD = headache; INF = infection; INSECTICIDE = delousing; MAGIC = disease of unidentified etiology (‘magical poisoning’); MAINT = health promotion, including failure to thrive; MAL = Malaria; NUT = nutritional supplement; OCC = ocular diseases; ORG = diseases thought to affect one particular organ; OTHER = unclear disease syndrome; PAIN = physical pain; POIS = envenomation or poisoning; sometimes this includes transnatural causation; PSYCH = psychiatric diseases or syndromes; REP = reproductive diseases including childbirth related issues; RESP = respiratory diseases; SKIN = dermal related diseases; often includes infectious disease; SWELL = swelling of whole body or part of the body; URINE = urinary conditions; WOUND = wound related diseases or syndromes

Route of Administration codes (RouteCode) as follows: O = oral; T = topical; I = inhalation; P_to_Plant = patient to plant transfer of blood

Mode of preparation codes (PrepCode) as follows: B = burned (smoke generation), C = cooked; D=decoction, H = heated, HR = heated then rubbed, M = masticated, MAG = magical, MS = masticated then spit on affected area(s), R = raw; S = succus (crushed), V = vapor

Plant part utilized codes (PartCode) as follows: R = Rhizome, L = Leaf, yL = young leaf, B = Bark, yShoot = young shoot

### Shared and unique plants

We found a number of plants were reported as used in common amongst these areas. Many plants had many overlaps in use, preparation, and disease (Table 1). However, among the plants identified to species level, only four species were reported in every survey: *Alstonia scholaris* (L.) R.Br., *Cassia alata* L.*, Passiflora foetida* L., and *Zingiber officianale* Roscoe. The number of plants unique to one or another of the four reports was surprisingly large in comparison to the previous reports [8, 9]. A total of 80 genera, of which 29 are identified to genus level and 51 to species level (see Table 2), were not shared between any of the four study areas.

**Table 2:** Plants not shared between the four study areas in East Sepik Province

BK	DK	GW	MS
*Albizia saman* (Jacq.) Merr. (BK 058/06)	*Ageratum conyzoides* (L.) L. (DK 38/05)	*Albizia procera* (Roxb.) Benth. (GW 09/04)	*Abelmoschus manihot* (L.) Medik. (MS 02/04)
*Cascabela thevetia* (L.) Lippold (BK 028/06)	*Angiopteris evecta* (G. Forst.) Hoffm. (DK 53/05)	*Bidens pilosa* L. (GW 40/04)	*Artocarpus altilis* (Parkinson ex F.A. Zorn) Fosberg (MS 23/04)
*Murraya paniculata* (BK 003/06)	*Areca catechu* L. (DK 02/05)	*Cerbera floribunda* K. Schum. (GW 12/04)	*Barringtonia asiatica* (L.) Kurz (MS 27/04)
*Premna serratifolia* L*.* (BK 052/06)	*Asplenium nidus* L. (DK 21/05)	*Clitoria terneata* L. (GW 91/04)	*Callicarpa longifolia* Lam. (MS 85/04)
*Tabernaemontana pandacaqui* Lam (BK 054/06)	*Bixa orellana* L. (DK 11/05)	*Gymnostoma papuana* (S. Moore) L.A.S. Johnson (GW 11/04)	*Calophyllum inophyllum* L. (MS 20/04)
	*Capsicum annuum* L. (DK 15/05)	*Hemigraphis reptans* (G. Forst.) T. Anders. ex Hemsl. (GW 70/04)	*Calotropis gigantea* (L.) Dryand (MS 32/04)
	*Carica papaya* L. (DK 34/05)	*Hydriastele costata* F.M. Bailey (GW 83/04)	*Caryota rumphiana* Mart. (MS 69/04)
	*Caryota mitis* Lour. (DK 26/05)	*Maclura cochinchinensis* (Lour.) Corner (GW 46/04)	*Casuarina equisetifolia* L. (MS 28/04)
	*Cheilocostus speciosus* (J. König) C. Specht (DK 20/05)	*Mangifera indica* L. (GW 93/04)	*Chrysopogon aciculatus* (Retz). Trin (MS 50/04)
	*Dendrocnide cordata* (Warb. ex H.J.P. Winkl.) Chew (DK 35/05)	*Neonauclea purpurea* (Roxb.) Merr. (GW 10/04)	*Cocos nucifera* L. (MS 78/04)
	*Homalium foetidum* (Roxb.) Benth. (DK 42/05)	*Pisonia longirostris* Teijsm. & Binn. (GW 32/04)	*Dendrocnide latifolia* (Gaudich.) Chew (MS 33/04)
	*Manihot esculenta* Crantz (DK 51/05)	*Solanum torvum* Sw. (GW 26/04	*Euphorbia tithymaloides* (L.) (MS 79/04)
	*Metroxylon sagu* Rottb. (DK 30/05)	*Sterculia shillinglawii* F. Muell. (GW 04/04)	*Ocimum basilicum* L. (MS 08/04)
	*Piscidia grandifolia* (Donn. Sm.) I.M. Johnst. (DK 31/05)		*Pandanus dubius* Spreng. (MS 30/04)
	*Planchonia papuana* R. Knuth (DK 45/05)		*Scaevola sericea* Vahl (MS 63/04)
	*Riedelia corallina* (K. Schum.) Valeton (DK 18/05)		*Schismatoglottis calyptrata* (Roxb.) Zoll. & Moritzi (MS 83/04)
			*Sida rhombifolia* L. (MS 76/04)
Identified to Genus only (Voucher)			
Christia sp. (BK 008/06)	Cinnamonum sp. (DK 54/05)	Aglaia sp. (GW 56/04)	Archidendron sp. (MS 01/04)
Clematis sp. (BK 049/06)		Asclepias sp. (GW 79/04)	Davallia sp. (MS 70/04)
Neonauclea sp. (BK 061/06)		Cissus sp. (GW 59/04)	Dillenia sp. (MS 81/04)
Phrynium sp. (BK 014/06)		Clerodendrum sp. (GW 87/04)	Graptophyllum sp. (MS 14/04)
Vanilla sp. (BK 012/06)		Desmodium sp. (GW 101/04)	Homalanthus sp. (MS 05/04)
		Mitracarpus sp. (GW 20/04)	Marattia sp. (MS 16/04)
		Papuechites sp. (GW 65/04)	Melastoma sp. (MS 36/04)
		Parsonia sp. (GW 29/04)	Villebrunea sp. (MS 13/04)
		Pouteria sp. (GW 41/04)	
		Semecarpus sp. (GW 55/04)	
		Smilax sp. (GW 99/04)	
		Spathiphyllum sp. (GW 33/04)	
		Tabernaemontana sp. (GW 76/04)	
		Tylophora sp. (GW 57/04)	
		Ursi sp. (GW 72/04)	

### Plant parts utilization, preparation, administration and diseases treated

**Fig. 1:**
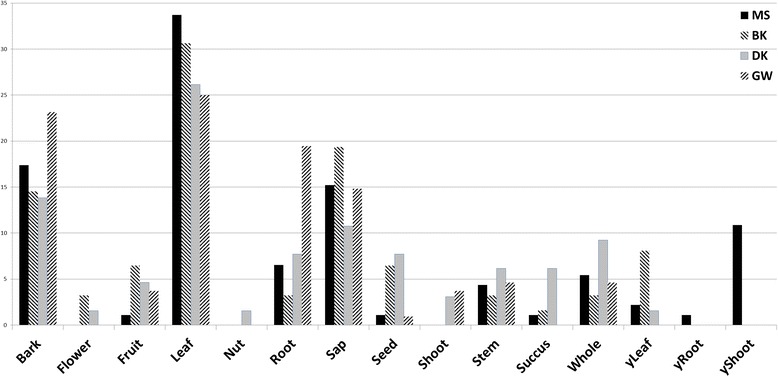
Traditional plant usage pattern by plant part utilized across four study areas in East Sepik province in percentile of total for each study area; *y* = young

In general the areas studied were similar in the relative utilization of plant parts (Fig. 1) with leaves predominating followed by bark and sap as next most common (with the exception of GW where roots were more commonly utilized than sap). The MS sample set reported a large number of young shoots/young roots stipulated for use in comparison to the other reports, where “young” was not specifically stipulated. The DK and GW reports only cited use of shoots. Only DK reported the medicinal use of nuts.

**Fig. 2:**
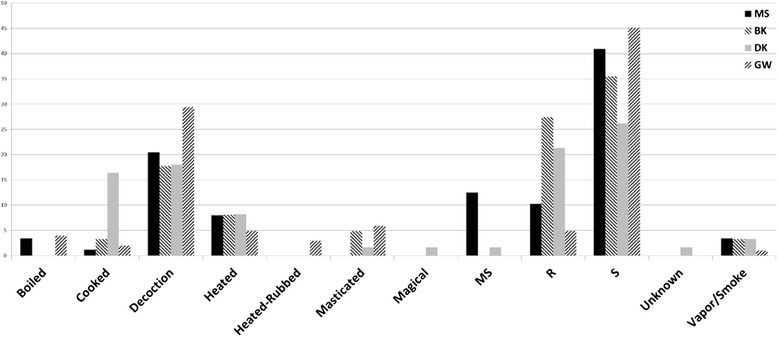
Method of preparation of plants for traditional medicines across four study areas in East Sepik province in percentile of total for each study area; MS = masticated then spit on affected area(s), R = raw; S = succus (crushed)

The method of preparation (Fig. 2) shows a similar pattern amongst the reports: use of succus (expressed juice) was most commonly reported, followed by decoction and direct application of the raw plant material. Usually direct application meant placing the material on a wound or skin ailment after minimal handling. Similarly all reports contain inhalation of smoke or vapor, heat treatment and cooking prior to utilization. DK reported a much higher frequency of cooking the material than the other areas. Boiling as a method of preparation was only mentioned in the MS and GW reports, while mastication (chewing) was reported in all except MS. Typically heating implies later consumption or preparation of steam for inhalation, however, in the GW report heating is a method to prepare the plant material prior to topical application (labelled HR—Heated-Rubbed). Another mode of preparation was mastication and spitting on the affected area. This was relatively common in the MS report and mentioned in the DK report, but not noted in the the other two areas. Only from the DK report is the reverse utilization of the plants reported, where in one instance *Homalium foetidum* (Roxb.) Benth. was utilized in a reverse-from-expected manner. In this case, the blood of the patient was placed under the bark of the tree with the expected result being a lessening of knee pain and strengthening of bones as the tree grew. This clearly implies a spiritual/magical connection of plant and patient.

**Fig 3:**
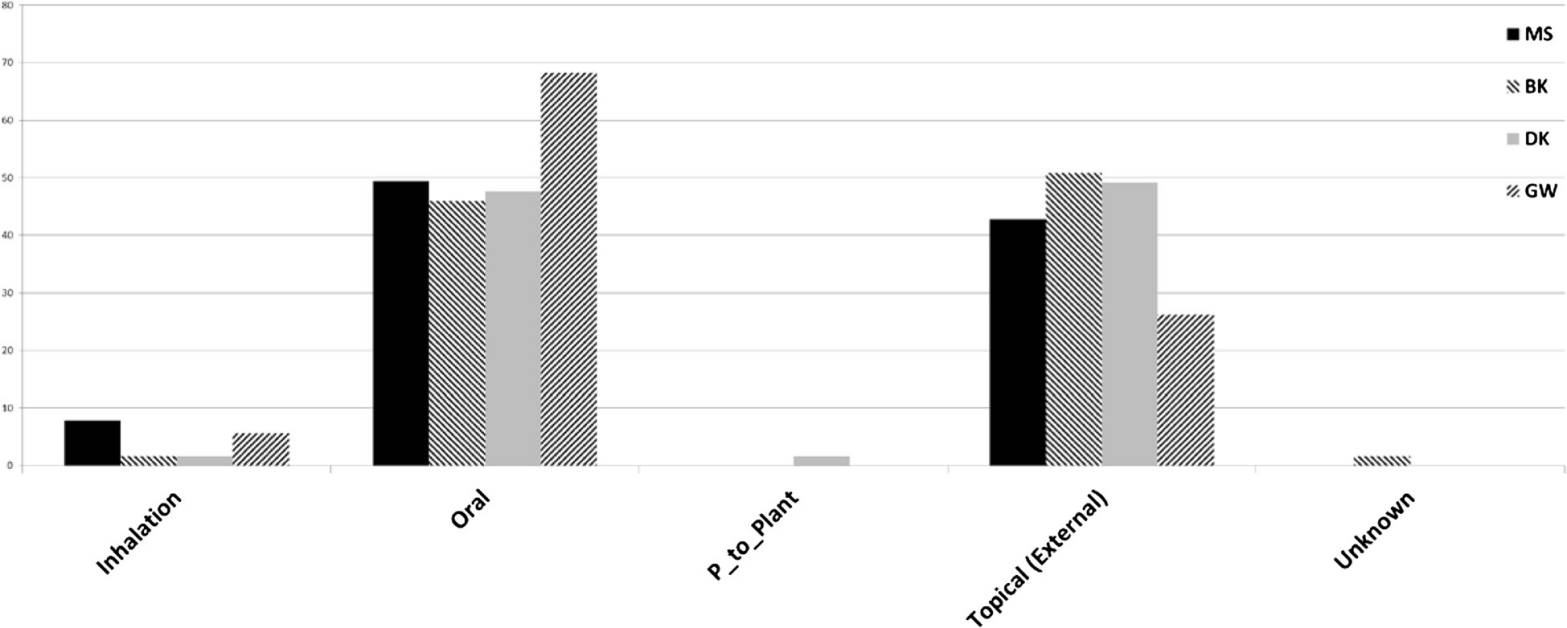
Mode of administration of plant based traditional medcines across four study areas in East Sepik province in percentile of of total for each study area; P_to_Plant = patient to plant transfer of blood

The routes of administration for plant based medicines reported by DK, BK and MS were about evenly divided between oral or topical routes (Fig. 3). The exception was the administration practices reported by GW where oral consumption outpaced topical application (3:2 ratio). Inhalation was reported only once for the DK and BK areas, and more frequently in the BW and MS areas. The lone outlier for route of administration was from the DK report in which patient material (blood) was transferred to the plant (as described above).

**Fig. 4:**
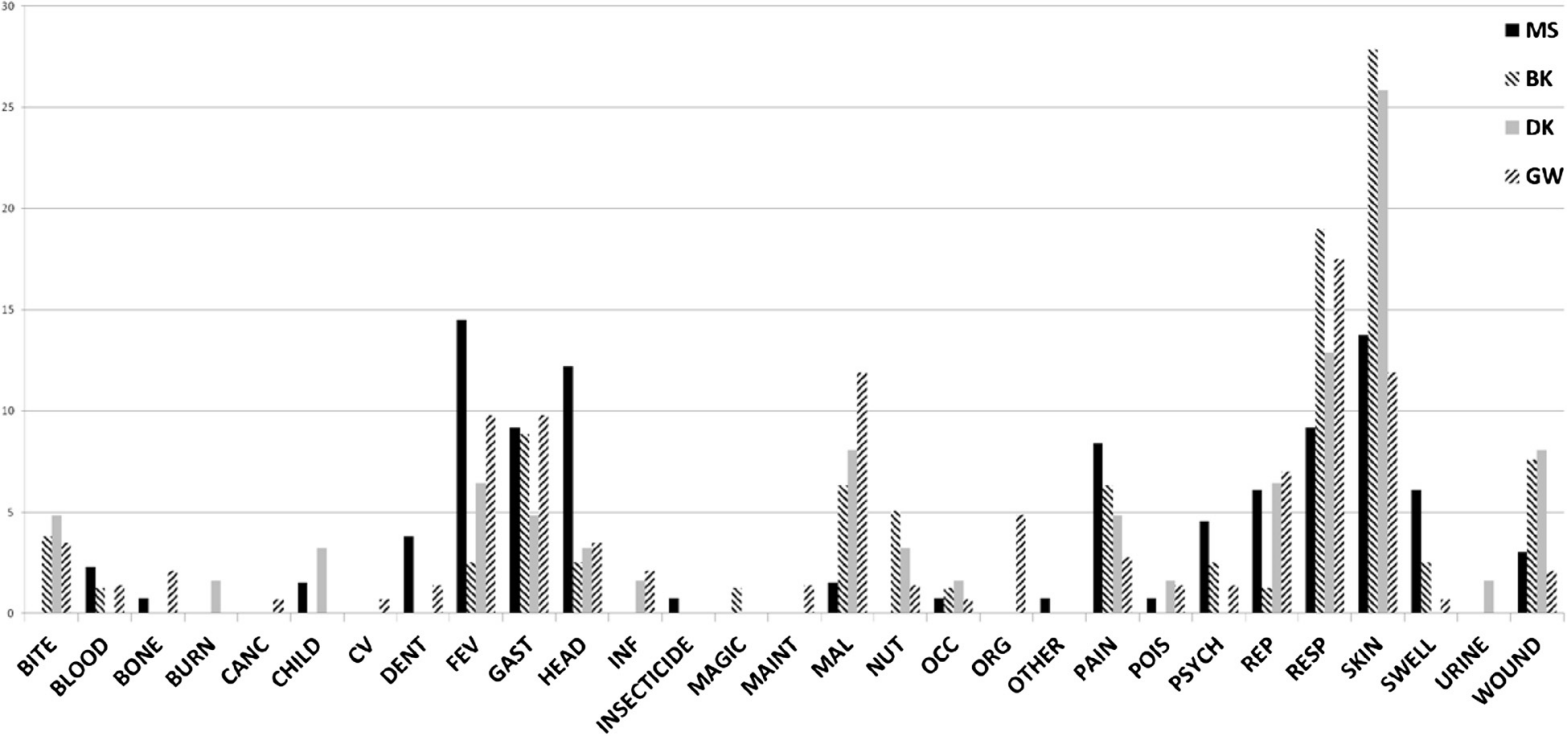
Coded ailments treated with plant based traditional medicines across four study areas in East Sepik province in percentile of of total for each study area; BITE = insect or snake bite; BLOOD = hematological issues including coagulation; BONE = bone related injury or disease; CANC = cancer; CV = Cardiovascular; CHILD = childhood disease; DENT = dental disease; FEV = fever; GAST = gasteroenterological disease; HEAD = headache; INF = infection; MAGIC = disease of unidentified etiology; MAINT = health promotion, including failure to thrive; MAL = Malaria; NUT = nutritional supplement; OCC = ocular diseases; ORG = diseases thought to affect one particular organ; POIS = envenomation or poisoning; sometimes this includes transnatural causation; PSYCH = psychiatric diseases or syndromes; REP = reproductive diseases including childbirth related issues; RESP = respiratory diseases; SKIN = dermal related diseases; often includes infectious disease; SWELL = swelling of whole body or part of the body; WOUND = wound related diseases or syndromes

Ailments treated (Fig. 4) with plant based medicines were categorized into 28 groups, sorted according to the target site, in order to to minimize possibly uncertain medical judgements or clinical misdiagnoses. Many described symptoms can likely accurately be ascribed to their appropriate causative diseases, but in the absence of independent clinical confirmation the decision was made to present the data in as unbiased a way as possible. Therefore, the category of “SKIN” contains both infections (e.g., “Grille”) and ectoparasitism (e.g., scabies); the category “REP” contains all sort of reproductive conditions, e.g., impotence, abortion, menstrual syndromes, contraception and fertility, etc. The exception to this method of categorization is malaria, which is generally well recognized throughout the Sepik. Overall, skin conditions were most frequently treated (73 instances), with respiratory conditions (60 instances), fever (39 instances), gastrointestinal conditions (36 instances) and malaria (29 instances) rounding out the top five conditions. The top five conditions in the respective reports were: for MS (fever—19, skin—18, headache—16, respiratory and gastrointestinal—12 reports each); BK (skin—22, respiratory—15, gastrointestinal conditions—7 wounds—6, and pain −5 instances): DK (skin—16, respiratory—8, malaria and wounds—5 instances each, and fever—4 instances): GW (respiratory—25, skin and malaria—17 each, gastrointestinal conditions and fever—14 instances each). The relative frequencies of ailments/conditions are presented in Fig. 4. Outlier conditions, those reported once and not reported in the other areas were urinary conditions (incontinence, URINE; and delousing, INSECTICIDE) from the MS report; use for burn conditions (BURN), magical poisoning (MAGIC) and child health improvement (CHILD) from the DK area; and cancer (CANC) and cardiovascular condition (CV) from the GW area.

### Most common families of plants used by healers interviewed

By far the most common genus was *Ficus* (11), followed by *Euphorbia* (7), *Piper* (6), *Plectranthus* (6), *Cassia* (5), *Passiflora* (5), and 4 instances each of: *Acalypha*, *Alpinia*, *Alstonia*, *Calamus*, *Crinum*, *Gnetum*, *Laportea*, *Merremia*, *Mucuna*, *Phyllanthus*, *Syzygium*, *Uncaria*, and *Zingiber*.

### Lesser known medicinal plant species of East Sepik

Those plants identified to the species level and not found in the Bougainville and Eastern Highlands reports were matched against our medicinal plants of PNG reference database, consisting of historical reports largely by Holdsworth and associates. The following plants were not described in the literature which the database encompasses: *Averrhoa carambola* L. (BK 039/06 & DK 01/05), *Campnosperma brevipetiolatum* Volkens Volkens. (DK 56/05), *Capsicum annuum* L. (DK 15/05), *Caryota mitis* Lour. (DK 26/05), *Cascabela thevetia* (L.) Lippold (BK 028/06), *Chrysopogon aciculatus* (Retz). Trin (MS 50/04), *Clitoria ternatea* L. (GW 91/04), *Curcuma longa* L. (BK 029/06), *Cycas rumphii* Miq. (BK 002/06), *Endospermum labios* Schodde (DK 40/05), *Endospermum formicarium* Becc. (GW 28/04), *Endospermum medullosum* L.S.Sm. (MS 89/04), *Erythrina merrilliana* Krukoff (GW 18/04 & MS 42/04), *Hydriastele costata* F.M. Bailey (GW 83/04), *Intsia bijuga* (Colebr.) Kuntze (DK 33/05 & GW 08/04 & MS 46/04), *Millettia pinnata* (L.) Panigrahi (GW 30/04), *Planchonia papuana* R. Knuth (DK 45/05), *Riedelia corallina* (K. Schum.) (DK 18/05), *Schismatoglottis calyptrata* (Roxb.) Zoll. & Moritzi (MS 83/04), *Sterculia shillinglawii* F. Muell. (GW 04/04), and *Tinospora arfakiana* Becc. (GW 82/04).

*Capsicum annuum* L. and *Curcuma longa* L. are commonly grown in many gardens across PNG, yet it was surprising to note the paucity of medicinal uses previously reported for PNG. *Ipomoea pes-caprae* (L.) R. Br. (BK 020/06 & MS 26/04) also did not appear to be part of the older literature, however, it was recently found to be used in the New Britain Province where the leaves are rubbed onto the skin affected by jelly fish stings [12]. The sap is used in the BK area for respiratory ailments, and the succus from the leaves is reported by MS to be used in Kairiru for fever/pain via oral consumption.

### Comparing East Sepik with Eastern highlands and Bougainville provinces

**Fig. 5:**
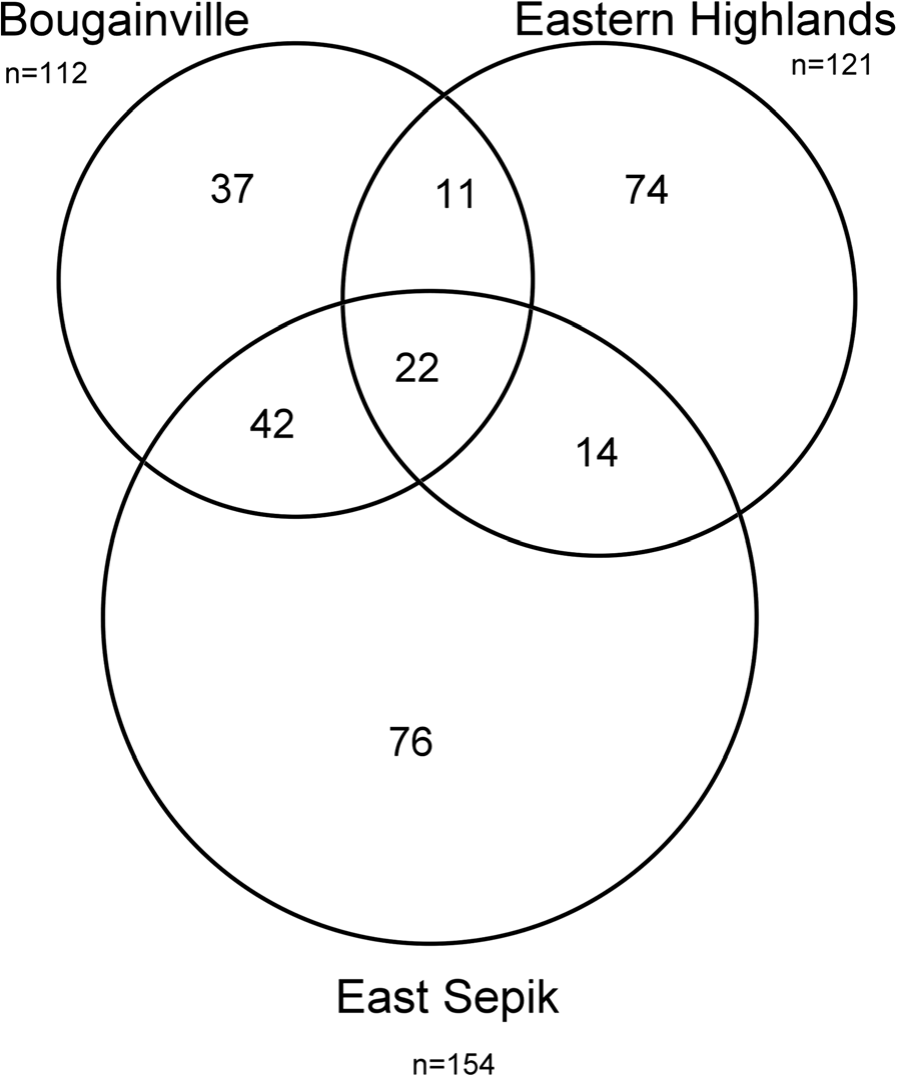
Venn diagram showing the total number (n) and overlap of plant genera utilized medicinally between Bougainville, East Sepik, and Eastern Highlands provinces

The combined dataset of the East Sepik, Eastern Highlands and Bougainville reports encompasses 276 plant genera, of which only 22 were reported in common from our other published data sets; Bougainville 112 genera, Eastern highlands 121, and East Sepik 154 genera (see Fig. 5). The frequency of shared genera is given in Table 3. The plant genera with the highest common use citations (> = 10) are Ficus sp. 29, Alpinia sp. 16, Piper sp. 15, Syzygium sp. 12 and Alstonia sp.11. The predominance of Ficus sp. is not surprising since Ficus represents a very large genus in PNG [13].

**Table 3:** Plant Genera in common utilized in Bougainville, Eastern Highlands and East Sepik Provinces

Genus	Bougainville	Eastern Highlands	East Sepik	Total
Ageratum	2	1	1	4
Alpinia	7	5	4	16
Alstonia	4	3	4	11
Aristolochia	1	1	2	4
Barringtonia	2	1	1	4
Ficus	11	7	11	29
Graptophyllum	1	1	1	3
Hemigraphis	1	2	1	4
Leucosyke	1	1	1	3
Litsea	1	1	2	4
Melastoma	1	2	1	4
Mucuna	3	1	5	9
Musa	2	1	2	5
Piper	4	5	6	15
Plectranthus	2	1	6	9
Psidium	2	1	2	5
Sida	1	1	1	3
Smilax	1	3	1	5
Syzygium	4	4	4	12
Uncaria	2	1	2	5
Zingiber	1	2	4	7

### Regional utilization of plants

Comparison of plants used medicinally in our published data sets to a general list of plants from the same regions allowed for an analysis of utilization preferences. Medicinally over- and under-represented plant families are given in Table 4, while medical plant utilization is given in Table 5. Comparison shows that the number of plant families significantly underutilized, when compared against the regional flora, breaks down as follows: in East Sepik (ES) province Poaceae are underutilized, while in the Eastern Highlands (EH) and Bougainville (BV) Orchidaceae are underutilized.

**Table 4:** Overrepresented and underespresented plants for each province when compared to the regional plant diversity as recorded in the PNG Plant Database

East Sepik (ES Reports vs PNG PlantDB (ES total flora)					
	# in PNG PlantDB	# in ES Medical Plants Reports	Inferior Credible Interval	Superior Credible Interval	Difference to interval (margin)
Total	2258	207	0.080	0.104	-
Overrepresented Families					
Araceae	13	7	0.289	0.770	0.184
Zingiberaceae	14	6	0.213	0.677	0.108
Marantaceae	3	2	0.194	0.932	0.090
Solanaceae	9	4	0.187	0.738	0.083
Euphorbiaceae	83	22	0.182	0.369	0.078
Convolvulaceae	13	5	0.177	0.649	0.072
Datiscaceae	1	1	0.158	0.987	0.054
Fabaceae	82	19	0.154	0.334	0.050
Gnetaceae	4	2	0.147	0.853	0.042
Davalliaceae	8	3	0.137	0.701	0.033
Lamiaceae	42	10	0.135	0.386	0.031
Anacardiaceae	18	5	0.126	0.512	0.021
Asteraceae	19	5	0.119	0.491	0.015
Menispermaceae	15	4	0.110	0.524	0.006
Piperaceae	15	4	0.110	0.524	0.006
Underrepresented Families					
Poaceae	106	3	0.010	0.080	0.028
Eastern Highlands (EH) vs PNG PlantDB (EH Total Flora)					
	# in PNG PlantDB	# in EH Medical Plants Reports	Inferior Credible Interval	Superior Credible Interval	Difference to interval (margin)
Total	3549	156	0.038	0.051	-
Overrepresented Families					
Ebenaceae	2	2	0.292	0.992	0.241
Winteraceae	2	2	0.292	0.992	0.241
Acanthaceae	12	5	0.192	0.684	0.141
Hypoxidaceae	1	1	0.158	0.987	0.107
Smilacaceae	7	3	0.157	0.755	0.106
Plantaginaceae	5	2	0.118	0.777	0.067
Lamiaceae	21	5	0.107	0.454	0.056
Araliaceae	17	4	0.097	0.476	0.046
Commelinaceae	2	1	0.094	0.906	0.043
Elaeagnaceae	2	1	0.094	0.906	0.043
Actinidiaceae	14	3	0.078	0.481	0.027
Asteraceae	103	13	0.076	0.204	0.024
Bignoniaceae	3	1	0.068	0.806	0.016
Casuarinaceae	3	1	0.068	0.806	0.016
Lecythidaceae	3	1	0.068	0.806	0.016
Symplocaceae	3	1	0.068	0.806	0.016
Onagraceae	9	2	0.067	0.556	0.016
Theaceae	9	2	0.067	0.556	0.016
Begoniaceae	10	2	0.060	0.518	0.009
Balsaminaceae	4	1	0.053	0.716	0.002
Caprifoliaceae	4	1	0.053	0.716	0.002
Icacinaceae	4	1	0.053	0.716	0.002
Oxalidaceae	4	1	0.053	0.716	0.002
Selaginellaceae	4	1	0.053	0.716	0.002
Usneaceae	4	1	0.053	0.716	0.002
Underrepresented Families					
Orchidaceae	191	1	0.001	0.029	−0.009
Bougainville (BV) Reports vs PNG PlantDB (BV Total Flora)					
	# in PNG PlantDB	# in BV Medical Plants Reports	Inferior Credible Interval	Superior Credible Interval	Difference to interval (margin)
Total	1524	154	0.087	0.117	-
Overrepresented Families					
Verbenaceae	3	3	0.398	0.994	0.280
Musaceae	2	2	0.292	0.992	0.175
Zingiberaceae	19	9	0.272	0.685	0.155
Gnetaceae	3	2	0.194	0.932	0.077
Arecaceae	19	7	0.191	0.592	0.074
Marattiaceae	6	3	0.184	0.816	0.067
Caricaceae	1	1	0.158	0.987	0.041
Xanthorrhoeaceae	1	1	0.158	0.987	0.041
Leeaceae	4	2	0.147	0.853	0.029
Fabaceae	53	12	0.135	0.356	0.018
Thelypteridaceae	9	3	0.122	0.652	0.004
Malvaceae	30	7	0.119	0.411	0.001
Underrepresented Families					
Orchidaceae	74	1	0.003	0.072	−0.015

*BS* Bougainville, *EH* Eastern Highlands, *ES* East Sepik, *PNG PlantDB* Papaua New Guinea Plant Database [10]

**Table 5:** Overrepresented and underespresented plants for each province when compared to the regional plant diversity as recorded in the UPNG Traditional Medicines Database

East Sepik (ES Reports vs UPNG TradMed DB					
	# in UPNG TradMed DB	# in ES Medical Plants Reports	Inferior Credible Interval	Superior Credible Interval	Difference to interval (margin)
Total	1176	203	0.152	0.195	-
Overrepresented Families					
Convolvulaceae	6	5	0.421	0.963	0.226
Arecaceae	10	7	0.390	0.891	0.195
Marantaceae	2	2	0.292	0.992	0.097
Apocynaceae	25	9	0.202	0.557	0.007
Underrepresented Families					
Verbenaceae	22	0	0.001	0.148	−0.004
Eastern Highlands (EH) vs UPNG TradMed DB					
	# in UPNG TradMed DB	# in EH Medical Plants Reports	Inferior Credible Interval	Superior Credible Interval	Difference to interval (margin)
Total	1176	147	0.107	0.145	-
Overrepresented Families					
Monimiaceae	2	2	0.292	0.992	0.147
Plantaginaceae	2	2	0.292	0.992	0.147
Winteraceae	2	2	0.292	0.992	0.147
Melastomataceae	7	4	0.245	0.843	0.100
Asparagaceae	5	3	0.223	0.882	0.078
Smilacaceae	5	3	0.223	0.882	0.078
Onagraceae	3	2	0.194	0.932	0.049
Pittosporaceae	3	2	0.194	0.932	0.049
Asteraceae	47	13	0.170	0.418	0.024
Phyllanthaceae	1	1	0.158	0.987	0.013
Caryophyllaceae	1	1	0.158	0.987	0.013
Chloranthoceae	1	1	0.158	0.987	0.013
Elaegnaceae	1	1	0.158	0.987	0.013
Oleaceae	1	1	0.158	0.987	0.013
Polygalaceae	1	1	0.158	0.987	0.013
Tiliaceae	1	1	0.158	0.987	0.013
Proteaceae	4	2	0.147	0.853	0.001
Underrepresented Families					
Euphorbiaceae	88	3	0.012	0.095	−0.012
Bougainville (BV) Reports vs UPNG TradMedDB					
	# in UPNG TradMed DB	# in BV Medical Plants Reports	Inferior Credible Interval	Superior Credible Interval	Difference to interval (margin)
Total	1177	146	0.106	0.144	meh
Overrepresented Families					
Arecaceae	10	7	0.390	0.891	0.246
Leeaceae	2	2	0.292	0.992	0.148
Rhizophoraceae	2	2	0.292	0.992	0.148
Thelypteridaceae	5	3	0.223	0.882	0.079
Zingiberaceae	23	9	0.221	0.594	0.077
Malvaceae	17	7	0.215	0.643	0.071
Salicaceae	1	1	0.158	0.987	0.014
Pteridaceae	1	1	0.158	0.987	0.014
Scrophulariaceae	1	1	0.158	0.987	0.014
Marattiaceae	7	3	0.157	0.755	0.013
Moraceae	38	10	0.150	0.421	0.006
Gnetaceae	4	2	0.147	0.853	0.003
Underrepresented Families					
None found					

*BS* Bougainville, *EH* Eastern Highlands, *ES* East Sepik, *UPNG TradMedDB* University of Papaua New Guinea Traditional Medicines Database [9, 25]

The number of plants overutilized varies (ES: *n* = 15; EH: *n* = 25 and BV: *n* = 12) but is relatively stable as percentage of plants found in the regional database at 0.66, 0.7 and 0.78 % for ES, EH and BV, respectively. East Sepik shares overutilization of Fabaceae, Gnetaceae and Zingiberaceae with Bougainville and overutilization of Asteraceae and Lamiaceae with Eastern Highlands, while Eastern Highlands and Bougainville share no overutilized plant families.

When the UPNG Traditional Medicines Database was used to assess utilization, the underrepresented plant families were the Verbenaceae in East Sepik and the Euphorbiaceae in the Eastern Highlands. No plant family met the *p* = 0.05 criterion in Bougainville, however, Euphorbiaceae was the top ranked underutilized plant family (data not shown). The number of overutilized plants is varied (ES: *n* = 4; EH: *n* = 17; BV: *n* = 12). Among the overused plant families East Sepik shared the Arecaceae with Bougainville. Several plant families reappear in this analysis, e.g., the Asteraceae and Winteraceae from the Eastern Highlands province and the Gnetaceae and Zingiberaceae in Bougainville. The statistical requirements of the comparison method resulted in some plant families appearing in the overutilization category represent a single report from the region for that plant family. This could not be avoided since the East Sepik reports are included in the UPNG Traditional Medicines Database total. As the PNG Medicinal Plant Database database grows in the future the stringency of the analysis will improve.

Traditional inspection of the information gathered yielded information about plants not widely used, poorly annotated or used for different ailments than those in locales where use of the plant is more common. Plants without annotation in the recent PNG Medicinal Plant Literature include:

*Alocasia cucullata* (Lour.) G. Don surprisingly did not yield any crossrefernces in the PNG database, even when using synonyms. It is used in Chinese medicine for snakebite, abscesses, rheumatism, and arthritis [14] and has recently been identified as containing anticancer compounds [15, 16].

*Averrhoa carambola* L. (starfruit) fruit is used for cuts and asthma in PNG, and also widely used throughout the world for a variety of ailments, seemingly only in India as antihemmoraghic [17].

*Caryota mitis* Lour. has no further medicinal annotation for use in PNG, but is used several Asian countries for a variety of ailments, e.g., against hemorrhoids, male sexual dysfunction, and rheumathoid arthritis in Bangladesh [17].

*Chrysopogon aciculatus* (Retz). Trin is used in the East Sepik for swelling. The plant is used in Ayurveda as a diuretic [17, 18].

*Clitoria ternatea* L*.*is used for infertility in PNG and similarly in Ayurveda, where fresh root juice in fresh goat milk is used for pregnancy [18], however, the plant is used for a dizzying array of conditions and ascribed activities [17].

*Endospermum medullosum* L.S.Sm. has been described previously as used against rheumatism [18], perhaps similar to the use against general body pain in the East Sepik.

Used as a contraceptive in the East Sepik, *Erythrina merrilliana* Krukoff reveals a dearth of information regarding medicinal uses. The plant is however known to produce toxic alkaloids [19].

*Gnetum gnemonoides* Brongn. yielded very little information as to medicinal use, but has been described to contain a variety of stilbenes [20].

*Hemigraphis reptans* (G. Forst.) T. Anderson ex Hemsl. is used in the East Sepik as the whole plant to treat centipede bite. The root is expressed into water to facilitate birth (speeding up delivery) on Vanuatu [21].

No medicinal use annotation was found for *Hydriastele costata* F.M. Bailey and therefore it may present one of the plants which is used very rarely for that purpose.

*Intsia bijuga* (Colebr.) Kuntze has annotations as a detoxicant and against diarrhea, toothache, adenopathy and swelling [22].

*Macaranga clavata* Warb. is used in East Sepik for skin infections, but has no recent mention in the literature for medicinal use. No scientific background information was located, hence this particular plant may be understudied. The same is also true for *Macaranga darbyshirei* Airy Shaw, used in the East Sepik as an antivenom, but not elsewhere mentioned for medicinal purposes.

*Pandanus dubius* Spreng. was not found to have any properly referenced medicinal annotations, but appears to have a fairly recent research record including discovery of two novel alkaloids, dubiusamines-A and dubiusamines-B [23].

*Piper mestonii* F.M. Bailey leaves used for fresh cuts and wounds do not seem to be described elsewhere. No biochemical investigation could be located in the Dictionary of Natural Products [24].

*Planchonia papuana* R. Knuth appears to be not used medicinally elsewhere. It is a timber tree and perhaps as such has not attracted attention; however, in an antiviral screen in our lab fractions from *P. papuana* exhibited anti-HIV activity [25].

*Plectranthus parviflorus* Willd., along with *Plectranthus blumei* (Benth). Launert, and *Plectranthus myrianthus* Briq. belong to a genus prominent for production of essentials oils [26] and with multiple annotations for antimicrobial activity, but do not seem to be described elsewhere in the PNG plant literature. The utilization of these plants for sores, ulcers and fresh cuts appear to be in line with the activities of chemicals found in *Plectranthrus* species [27].

*Riedelia corallina* (K. Schum.) Valeton, in the Zingiber family, is used for menstrual cramps, but seems to be otherwise undescribed for medicinal uses elsewhere.

The leaves of *Schismatoglottis calyptrata* (Roxb.) Zoll. & Moritzi are used in East Sepik to treat skin sores. No other mention was found in the PNG literature. The stems of *Schismatoglottis calyptrata* (Roxb.) Zoll. & Moritzi are however used in Chinese medicine for treatment of lumbago and arthralgia [18].

*Sterculia shillinglawii* F. Muell. has no previous annotation for PNG, but is known to be used in the Solomon island as a tonic and to reduce fever [18].

*Tinospora arfakiana* Becc. likewise lacks further medicinal descriptions from PNG and does not seem to have been studied from any other area, making it a potentially understudied plant.

*Uncaria lanosa* var. *appendiculata* (Benth.) Ridsdale was mentioned twice in the reports and in both instances to treat fever, but also gastrointestinal diseases, malaria, and malnutrition. No other mention for ethnomedical use could be located from PNG or other locales. However, a recent publication hints at a potential anti-depressant effect of ethanolic extracts of *Uncaria lanosa* var. *appendiculata* (Benth.) Ridsdale [28].

*Uncaria orientalis* Guillaumin, used to treat shortness of breath in the East Sepik, lacks pharmacological annotation, but has been investigated extensively biochemically [29, 30].

## Conclusions

This report shows that in the East Sepik province of PNG the patterns of plant usage for medicinal indications is highly varied. This is true even though many of the same plants are used in ethnologically distinct regions. There is a tendancy for widely used plants to be used for multiple diseases, often with differing preparation of the parts utilized and differing modes of administration. One such example is *Alstonia scholaris* (L.) R.Br. which shares only the route of administration between all areas. Regardless, plants not previously documented as being used medicinally can still be uncovered, e.g., *Cascabela thevetia* (L.) Lippold, a plant known to contain highly toxic cardiac glycosides [31] and *Dendrocnide cordata* (Warb. ex H.J.P. Winkl.) Chew cannot be found as being used medicinally, however, toxicity from leaves, which are used in East Sepik, has been documented [18].

Comparison of plant utilization across study areas can likewise uncover plants which share use. A good example is the genus *Alpinia*, for which gasteroenterological, respiratory and reproductive use are cited for Bougainville. In the Eastern Highlands it is used for gasteroenterological and respiratory conditions. In the East Sepik it is also usedfor respiratory conditions. *Alpinia* is in the ginger family, widely used culinarily and medicinally around the world, with traditional medicinal uses for several of the described symptoms.

Likewise, dissemination of knowledge of useful phytomedicinal practices amongst areas that share key flora may aid health practices in those areas. In any case, further studies and phytochemical analyses need to be completed before addition of plants to the pharmacopeia for PNG (a goal of the National Policy for Traditional Medicne in PNG). The UPNG Traditional Medicines Database, while still being populated with data, can already be utilized to show correlations and extract lead information for targeting certain plants for further study. Further enhancements and perhaps adaptation of other data sources (e.g., the PNG Plant Database with up-to-date plant nomenclature) would drive statistical discovery of medicinally neglected plant genera. It is shown here that transregional comparisons are possible, but require careful recoding of previous reports and standardization of database entries and terminology.

Analysis of frequency of use of plant families in the medical tradition points to certain biases. This can ultimately be useful in targeting plants for biochemical investigation. However, if the desired outcome of the ethnobotany endeavor is to highlight useful plants for the pharmacopeia, then finer grained data is needed in order to dissect the wealth of information gathered, (e.g. precise geographic location including environmental conditions, etc.). Annotation with biochemical information, conservation status, toxicity data would yield utility for a more diverse set of scientists. To this end the diverse efforts of PNG botany, ethnobotany, ethnopharmacology and plant conservation need to collaborate more rigorously to define useful interfaces for each other’s data needs. Nevertheless, we have been able to successfully show that medicinal plant use in terms of families utilized in the East Sepik resembles Bougainville provinces more than it does the Eastern Highlands. Future work with larger data sets will address whether such similarities are due to similarities of available flora or other causes.
